# Fall armyworm from a maize multi-peril pest risk perspective

**DOI:** 10.3389/finsc.2022.971396

**Published:** 2022-12-19

**Authors:** Senait D. Senay, Philip G. Pardey, Yuan Chai, Laura Doughty, Roger Day

**Affiliations:** ^1^ GEMS Informatics Center, University of Minnesota, Saint Paul, MN, United States; ^2^ Department of Plant Pathology, University of Minnesota, Saint Paul, MN, United States; ^3^ Department of Applied Economics, University of Minnesota, Saint Paul, MN, United States; ^4^ CABI, Nosworthy Way, Wallingford, United Kingdom

**Keywords:** maize, multi-peril pest risk, pest risk modeling, fall armyworm, *Spodoptera frugiperda*

## Abstract

We assembled 3,175 geo-tagged occurrences of fall armyworm worldwide and used that data in conjunction with information about the physiological requirements of the pest to spatially assess its global climate suitability. Our analysis indicates that almost the entire African maize crop is grown in areas with climates that support seasonal infestations of the insect, while almost 92% of the maize area supports year-round growth of the pest. In contrast, rich-country maize production largely occurs in temperate areas where only 2.3% of the area may allow the pest to survive year-round, although still subject to worrisome seasonal risks. This means the African maize crop is especially susceptible to damaging infestation from fall armyworm, on par with the risk exposure to this pest faced by maize producers throughout Latin America. We show that the maize grown in Africa is also especially vulnerable to infestations from a host of other crop pests. Our multi-peril pest risk study reveals that over 95% of the African maize area deemed climate suitable for fall armyworm, can also support year-round survival of at least three or more pests. The spatial concurrence of climatically suitable locations for these pests raises the production risk for farmers well above the risks posed from fall armyworm alone. Starkly, over half (52.5%) of the African maize area deemed suitable for fall armyworm is also at risk from a further nine pests, while over a third (38.1%) of the area is susceptible to an additional 10 pests. This constitutes an exceptionally risky production environment for African maize producers, with substantive and complex implications for developing and implementing crop breeding, biological, chemical and other crop management strategies to help mitigate these multi-peril risks.

## 1 Introduction

Outbreaks of fall armyworm (FAW) (*Spodoptera frugiperda*, J. E.Smith (Insecta: Lepidoptera: Noctuidae) were first observed in southwest Nigerian maize fields in January 2016, and shortly thereafter in Benin, Togo and São Tome and Principe (1). Since then, the pest’s African presence has gained considerable professional and practical attention (e.g. ,^2^–^5^) and substantial support from the international donor and research communities (e.g. 6–8). Reported occurrences have now spread well beyond the initial sightings in West Africa to include East African countries (Ethiopia, Kenya and Tanzania, see 5) and elsewhere on the continent. Prasanna et al. (4, page 2) noted more than 40 African countries with occurrences of the pest.

Fall armyworms have long been an established and costly problem for U.S. farmers, affecting maize, pasture, rice, sorghum, cotton, and many other crops since the mid-1800s. Summarizing the relevant molecular evidence, du Plessis et al. (9, p.1) observed, “Two distinct genotypes of *S. frugiperda*, each with its host preferences and some minor differences in biology have been reported (10–12). These genotypes have since been characterised as comprising sister species (13). The “maize/corn strain” prefers to attack maize and sorghum, while the “rice strain” prefers to attack rice and certain forage grasses (14–16). Both the maize and rice strains have been found in Africa (13), and molecular evidence shows that the fall armyworms found in Africa are genetically similar to the strains found in the U.S. (13, 17). There is also evidence for the possibility of introductions from Asia (18) as well as Latin America (19). The insect is still problematic for many farmers, especially those in the southern states of the U.S. and throughout Latin America and the Caribbean (20, 21), with seasonal outbreaks also found in southeastern Canada (22, p. 71). As the insect does not hibernate, it cannot survive cold winters, but it does overwinter in sub-tropical and tropical locales and in the moth stage of its lifecycle can migrate over long distances—upwards of 1,900 km by stepwise migration with the aid of wind currents (23, 24) to damage crops grown in temperate regions (25).

Notwithstanding the recent attention given to infestations of *S. frugiperda* in Africa, other armyworm species have long been a scourge of African maize farmers. Writing in 1975, Brown and Dewhurst 26 identified 23 species of armyworm (genus *Spodoptera*) worldwide, and discussed in detail eight species that occurred in Africa (and for some at least, elsewhere in the world). Pogue, (22) more recent phylogenic examination of the genus reports 30 species worldwide, half of which are considered pests. Of note is the Africa armyworm, *Spodoptera exempta* (Walker), which Brown and Dewhurst (26, p. 245) asserted as “… the most important armyworm in Africa [at that point in time], and in the scale of its attacks [on agriculture] one of the most severe in the world.” Haggis (27, p. 1) noted that African armyworm “… is recorded very widely in Africa south of the Sahara” and that it also occurs “…intermittently through the oceanic countries of South East Asia and the Pacific as far east as Hawaii, but not in the Americas.” Rose et al. (28, p.1) observed that “…the African armyworm has gained notoriety as a pest species second only to locusts.”

But armyworms are not the only pests plaguing African farmers, or the crop breeders and agronomic management professionals who serve them. For those species that cause crop damage, gauging which particular pest or complex of pests to prioritize over others is complicated. These yield-reducing and risk-increasing outcomes are both difficult to quantify—especially over large geographical areas—given the complex pest-host-environment interactions in play and the lack of relevant data. These processes involve plant-pest interactions that are both spatially sensitive and time-dependent, and involve a multitude of interactions among the crop varietal and management choices made by farmers as well as the variable, longer-run climate and shorter-run weather factors they face (29). Placing an economic value on the production-increasing and risk-reducing outcomes associated with controlling a given pest is a critical component of any priority setting exercise but represents only part of the puzzle. For example, in taking preemptive action to mitigate the effects of fall armyworm—e.g., choosing to plant a particular maize variety that has (at least some) resistance to fall armyworm—farmers are making decisions based on their perceptions of the likelihood of an outbreak of fall armyworm on their particular fields in the coming season, the severity and extent of the occurrence, and the crop losses that may arise. However, what may be an economically rational choice ex ante (prior to the growing season when procuring seed), may look less than optimal in retrospect if pre-emptive mitigation costs were incurred for a season where fall armyworm was of little or no crop consequence. The divergence between the farmer’s ex ante (pre-season) and ex post (post-season) cost-benefit calculus would be even more pronounced if the farmer prepared for fall armyworm but incurred losses from another pest.

Historically, most of the scientific and farming focus has been on individual species that act to undermine crop yields or quality. In reality, multiple pests may be, and typically are, problematic for farming, which Koo and Pardey (30, p.7) dub a multi-peril pest problem. To complement and extend prior work regarding the problems fall armyworm poses for maize farmers worldwide, in this paper we place this pest within a multi-peril pest risk perspective. Before doing so, we briefly review the existing spatially-explicit evidence on the global habitat suitability for the survival and propagation of the pest, and compare that with our own climate-suitability assessment based on newly compiled geo-coded occurrence data. We then draw on climatic suitability data reported for 11 other high risk biotic threats of maize (31–38) to investigate the multi-peril risk faced by maize farmers around the world from fall armyworm plus these 11 other pests. Our pest co-occurrence evidence is juxtaposed against estimates of the global location of maize production to help gain a more encompassing perspective of the risks posed for maize producers by fall armyworm vis-à-vis other problematic pests. We conclude with a discussion of this new multi-peril pest risk evidence from both a global and an African perspective.

## 2 Material and methods

### 2.1 Revisiting the climatic suitability for fall armyworm

As Mitchell (21, p. 452) observed, “Philip Luginbill’s 1928 treatise on the fall armyworm, *Spodoptera frugiperda* (J. E. Smith), long has been considered the source for authoritative information on the biology and dynamics of this pest.” Luginbill’s treatise provides a significant amount of information on the pest’s phenology, including evidence on locations where the climate is deemed suitable for the year-round survival and propagation of fall armyworm, and the geographical extent of seasonal outbreaks of the pest across the United States (see, e.g., 20). More recent, and more formal, quantitative approaches to assessing the geographical pattern of fall armyworm risk include studies by du Plessis et al. (9) Ramirez-Cabral et al. (39) Early et al. (40) Baloch et al. (41) Maino et al. (42), and Timilsena et al. (43).

Ramirez-Cabral et al. (39) used two general climate circulation models to assess the prospective changes in infestation risk from projected changes in climate over the decades ahead. For their climate suitability assessment, Early et al. (40) compiled data for 876 presence locations and drew on eight (essentially correlative) modeling techniques to create an ensemble species distribution model that they used to predict a global climate suitability for fall armyworm. Their ensemble forecasting approach identified areas in Africa that had not yet reported the presence of the pest at the time of publication, using models that identified susceptible new areas by inferring from similarities in their climate to the conditions prevailing in previously infested areas. du Plessis et al. (9) reviewed the phenological (temperature and moisture) conditions suitable for pest growth and propagation and used the CLIMEX model (44, 45) along with occurrence points clustered mainly in the United States and the maize triangle of South Africa to conduct a spatially-explicit assessment of the climate suitability of fall armyworm worldwide. Timilsena et al. (43) also used a CLIMEX model and assembled 2,968 presence locations to update the modeled climate suitability of fall armyworm worldwide, while Baloch et al. (41) used a MaxEnt model to produce a global correlative climate suitability prediction for FAW. Maino et al. (42) examined the risk from FAW occurrence throughout Australia by a) assessing the potential suitability of a geographic area in terms of the physiological requirements of FAW, and b) accounting for the seasonal occurrence of the pest in relation to its dispersal from locations that from a physiological perspective could sustain FAW year round. Another recent regional study is that of Fan et al. (46), who used a logistic regression analysis on empirical physiological data and occurrence data for select regions within China to identify the spatial extent of FAW within China.

Here we refreshed the modeled evidence, motivated, in part, by reports that FAW is now overwintering in subtropical ranges both in the U.S and China (47, 48), and, concordant with these in-field findings, results from the phenological experiments conducted by Valdez-Torres et al. (49) regarding the minimum thermal threshold for FAW on maize. To accommodate these new lab and in-field findings, as described below we lowered the minimum threshold temperature parametrization in our CLIMEX model, which in conjunction with the expanded set of occurrence data used to calibrate our model, enabled us to update the spatial climate suitability extent of the pest relative to the prior modeled evidence. We give special attention to the spatial extent of the pest in newly invaded areas, where the elastic adaptation of FAW (50) allows the pest to occupy areas distinct from the native areas where the pest has long been problematic. In the **Supplementary Material** (**Supplementary Section 1**) we reveal measurable differences among the models, which become more pronounced in smaller spatial extents (e.g., countries versus regions or the world), particularly in the sub-tropical regions of the world where the pest has recently been observed in the field and can survive based on empirical lab results.

### 2.2 Occurrence data

To reassess where climate is deemed suitable for the seasonal growth and year-round survival of fall armyworm, we first compiled a database of reported geo-coded occurrences of the pest, which we sourced from the Global Biodiversity Information Facility (51), plus other literature and data sources (**Table 1**). From our initial compilation of 4,497 observations, we set aside observations that had spatially inconsistent records—i.e., when the location descriptions in the metadata mismatched the reported geographic coordinates—or were duplicated across our various sources. This left us with 3,175 observations for inclusion in our initial climate-suitability assessment.

**Table 1:** Data source details for fall armyworm occurrences.

Sources	Observations			
	Original	Cleaned	Spatial duplicates removed (10 arc minute)	Remarks
*(count)*				
GBIF (21)	3,698	2,779	413	Points without GeoRef and with spatial inconsistency were removed
Sisay et al. (5)	29	29	29	East African FAW locations
Early et al. (40)	761	358	358	Points already in GBIF were removed. This data source added mostly novel South American locations (that were not reported in GBIF)
ACAPS (52)	9	9	9	Locations for Uganda, Zimbabwe, DR Congo, Malawi and Zimbabwe
**Total**	**4,497**	**3,175**	**809**	

To align with the spatial resolution of the climate data used in the CLIMEX model, observations that constitute duplicates within the 10-arc minute climate grid were removed to avoid mis-estimation of model accuracy during evaluation.

When modeling the climate suitability of a pest, it is best to have data that are representative of the range of climates in which the pest may propagate and persist, or not.^1^ Observational data that are tightly clustered in a certain spatial (and thereby, often, narrow and perhaps unrepresentative agroecological) range can result in overfitting a niche analysis model. In our case, 2,529 (79.6%) of the observations are from North America, 508 (16%) from Central and South America (and the Caribbean), 110 (3.5%) from sub-Saharan Africa, and 28 (0.9%) from Asia-Pacific (**Supplementary Table 2**). Notably, 933 (29.3%) of our observations lie within “native” (i.e., historically occurring) tropical areas (53, p. 554) where the phenology of the pest indicates the possibility of year-round survivability. The remaining 2,242 (70.6%) observations are from a) historically seasonally invaded temperate areas where the insect may invade and propagate but is unlikely to survive over winter (specifically areas in North America), or b) from newly invaded, mainly but not always tropical, areas in Africa and elsewhere. Among the 2,242 occurrences that cover the invaded range, 2,104 are in North America, to the north of the pest’s persistent native range, while 138 observations are from the rest-of-the-world, mainly in sub-Saharan Africa, India and China (**Supplementary Table 2**). Most of the observations (1,882, around 60%) were for pest sightings after 2010, of which over 70% occurred in the recently invaded ranges in sub-Saharan Africa, India, China, Australia and the historical seasonally invaded areas of North America

### 2.3 Correlative environmental niche analysis

To hone in on the climate preferences for fall armyworm, we began by investigating the climate concordance between occurrences in more recently invaded areas versus its “native” or historical range of occurrence. For the niche analysis, we used a bioclimatic dataset that has 39 variables drawn from environmental predictors like temperature, precipitation, moisture and solar radiation (54) and topographical predictors such as slope, aspect and hillshade that we generated from a digital elevation model (DEM) downloaded from the WORLDCLIM data portal (55, 56) (see **Supplementary Table 1**).

We used a two-step process to identify the variables deemed most important in accounting for the environmental niches of both the native and invaded ranges of fall armyworm. First, we carried out a variable selection procedure using a non-linear random forest approach (57) that reduced our variable set from 39 to 13 variables. Second, we carried out a PCA analysis with the smaller set of variables to generate the two principal components required to visualize the observed data in a bioclimatic representation of the niche space. These principal components represent artificial orthogonal axes derived from a linear combination of the values of the selected 13 bioclimatic variables at the locations of the observed occurrences.

For comparison we also performed a Principal Component Analysis (PCA) on the entire set of bioclimatic variables (n=39). We opted for a variable selection method that resulted in better environmental niche definition, based on the amount of variance accounted for by respective principal components.

The variable selection and PCA analyses were carried out using the free software R (58) version 4.1.0. The package randomForest (59) was used to conduct the variable selection, and an R-based niche modeling framework EcoSpat (60) was used to undertake the environmental niche analysis. Spatial data pre-processing and mapping were done using ArcGIS version 10.1 (61).

### 2.4 Process-based potential distribution modeling using CLIMEX

In the context of a CLIMEX model, the DV0 (i.e., lower temperature threshold) value is especially relevant in delineating the agroecological (and thus geographical extent) of a pest’s climate-suitability range. In calibrating their CLIMEX models, du Plessis et al. (9) Ramirez-Cabral et al. (39), and Timilsena et al. (43) set their DV0 values equal to 12.0°C. Based on the recent field occurrence observations gleaned from Osabutey et al. (48) for the U.S. and Yang et al. (47) where the temperature is as low as 9°C, coupled with Valdez-Torres et al. (49), lab findings that the minimum thermal threshold for FAW on maize was 8.7°C, we opted to set our DV0 value at 8.7°C. Thus, our lower limit was used to capture these subtropical ranges and ensure we did not underestimate the current risk coverage. Drawing from other model parameters reported by du Plessis et al. (9) and Early et al. (40), and aligning the modeled spatial extent with our expanded set of observational data, resulted in the final parametrization reported in **Supplementary Table 3**. To avoid over estimating model accuracy when spatially aligning the suitability results against the observed occurrences, we removed 2,366 occurrences that are redundant within the 10-arc minutes spatial resolution used in our analysis, leaving us with 809 unique occurrences that concorded with the spatial scale of the climate data used to run CLIMEX (**Table 1**).

Natural climate can be altered by agricultural operations. Notably the moisture regime affecting pest growth can be dramatically influenced by irrigation. To account for this possibility, two climate suitability projections were produced, one based on a rainfed scenario, the other based on an irrigation scenario involving a top-up of 2.5 mm of irrigated water per day throughout the year (45). Spatially explicit irrigation location data from You et al. (62) were used to identify the locations of irrigated areas as criteria to choose EI/GI values from either the rainfed or the irrigation CLIMEX scenarios to produce a mashed-up climate suitability map that addresses the potential to underestimate suitability in dry areas where the climate is moderated through irrigation. The CLIMEX model makes it possible to distinguish between locales where the climate is such that the pest poses a seasonally variable risk (designated as an annual Growth Index, GI) versus those locales where the pest is likely to persist year-round (designated as an Ecoclimatic Index, EI) at each geographical location—in this case a 10-arc minute geo‐referenced grid cell, roughly 18.5 x 18.5 kms resolution at the equator (63).

### 2.5 Multi-peril risk assessment

To gain a multi-peril perspective on the pest risks faced by maize farmers in sub-Saharan Africa and elsewhere in the world, we draw on estimates of the geographical extent and spatial concordance of climate-suitable locales for a portfolio of 12 maize pests. For FAW the updated climate suitability model from this study was used. Source details of the modeled climate suitability data for the other 11 maize pests was given above. This portfolio includes eight insects, three nematodes, and a parasitic weed (**Table 2**). Each of the CLIMEX projections of the 12 maize pests were first converted from tables with coordinate information into raster datasets. To spatially align the pest and maize geographies, the 10-arc minute grids obtained from the CLIMEX model were downscaled to a 5-arc minute resolution (roughly a 10 km grid at the equator) to concord with the gridded maize production data sourced from You et al. (62). Downscaling to a 5-arc minute grid resulted in GI and EI estimates for a total of 820,517 pixels spanning the entire cropped area of the world, from which we spatially parsed out the 561,268 pixels representing the location of maize production according to You et al. (62).

**Table 2:** Selected maize pests.

Pest		
Scientific name	Common name	Primary Crop Host
*Busseola fusca*	Maize stalk borer	Maize
*Cicadulina mbila*	(South African) maize leafhopper	Maize, rice, wheat, oats, barley, rye, finger millet, sorghum, and sugarcane, and wild grass species
*Chilo partellus*	Spotted stalk borer	Maize
*Diabrotica virgifera*	Western corn rootworm	Maize
*Frankliniella williamsi*	Corn thrips	Maize, cassava
*Hirschmanniella oryzae*	Rice root nematode (RRN)	Rice, maize, sugarcane, wheat, grasses, and sedges
*Heterodera zeae*	Nematode	Generalist on poaceae, especially maize, but also wheat, oats, barley etc.
*Meloidogyne graminicola*	Rice root-knot nematode	Generalist - maize, wheat, rice, sugar cane, etc.
*Ostinia nubilalis*	European corn borer	Maize
*Striga asiatica*	Asiatic witchweed	Maize
*Spodoptera exigua*	Beet armyworm	Generalist, beans, maize, peas, potato, tomato, oilseeds
*Spodoptera frugiperda*	Fall armyworm	Maize, rice, sorghum, cotton, sugarcane, wheat and other multiple crop and non-crop plants

Source: Compiled by authors from CABI (2021), and various other sources.

## 3 Results

### 3.1 Fall armyworm environmental niche

The two principal components that were derived from the full set of 39 variables accounted for only 63.2% of the total variance within the environmental values where fall armyworm was reported to occur. However, the PCA carried out on a smaller number of variables following a variable selection procedure using a non-linear random forest approach (57) reduced our variable set from 39 to 13 variables and performed much better. This two-step procedure increased the explanatory power of the two principal components to 87.9%.

The 13 top ranked “explanatory” variables that emerged from our random forest approach are tabulated in **Table 3**. The variables are grouped into two categories (temperature versus solar radiation), within which they are ranked in descending order according to their median absolute deviation (MAD). The MAD is a measure of how much error increases in the correlative model when a given variable is left out of the analysis, hence a higher number indicates increased “explanatory” importance for the variable. The PC1 and PC2 entries in the columns labeled PC1 and PC2 represent the loadings or coefficients for each of the relevant variables whose linear combinations constitute the respective principal components (PC1 and PC2) used to characterize the bioclimatic space in which fall armyworm occurrences are reported. Thus, the loadings or coefficients indicate the correlation between the original variables and the transformed principal components.

**Table 3:** Highly ranked bioclimatic variables used for the fall armyworm niche analysis.

Variables		MAD	Coefficients	
ID	Description		PC1	PC2
Temperature based predictors				
BIO 4	Temperature seasonality (C of V)	0.041	-0.329	-0.145
BIO 11	Mean temperature of coldest quarter (°C)	0.040	-0.309	0.051
BIO 9	Mean temperature of driest quarter (°C)	0.030	0.317	0.118
**BIO 6**	**Min temperature of coldest week (°C)**	0.029	**0.345**	0.011
BIO 3	Isothermality	0.028	0.335	-0.037
BIO 1	Annual mean temperature (°C)	0.025	-0.086	-0.421
BIO 7	Temperature annual range (°C)	0.019	-0.343	-0.070
Solar radiation based predictors				
BIO 26	Radiation of warmest quarter (W m^-2^)	0.046	0.282	-0.233
BIO 21	Highest weekly radiation (W m-2)	0.041	0.164	-0.466
BIO 24	Radiation of wettest quarter (W m^-2^)	0.035	0.328	-0.073
BIO 23	Radiation seasonality (C of V)	0.024	0.347	-0.017
BIO 20	Annual mean radiation (W m-2)	0.022	-0.055	-0.500
**BIO 22**	**Lowest weekly radiation (W m^-2^)**	0.019	-0.048	**-0.502**

We used the rule of thumb (namely the square root of one over the number of variables used in the analysis, i.e., 0.28) to delineate our cutoff value for designating “high-ranked” coefficients (loadings). The variable with the highest correlation with each of the principal components is highlighted in bold. The Isothermality variable, BIO 3, indicates temperature evenness throughout the year. It was derived as Bio 2/Bio 7 (see **Supplementary Table 1**). Bioclimatic variables absent units are dimensionless indices (54).

In prior work, Garcia et al. (64) established that ambient temperature, which affects the body temperature and hence developmental rate, of the insect was an important determinant of its spatial extent. Another set of variables that fall within our top ranked group of predictors indicate different dimensions of solar radiation, which is correlated with temperature variations (65). Aside from just being a proxy for temperature, Haynes et al. (66) reported solar radiation could be a strong predictor for the outbreak of defoliating insects (like fall armyworm), which they showed using long-term data tended to follow severe droughts. In their analysis, drought severity was more closely correlated with solar radiation than temperature. All of our highly ranked variables either directly or indirectly reflect different dimensions of temperature and solar radiation, specifically dimensions that are indicative of various bioclimatic thresholds that delineate the spatial distribution of fall armyworm.

Some of the variables, notably BIO 4, BIO 3, BIO 23 and BIO 7, are indicative of seasonal variation in temperature, and thus associated with climate attributes that sustain the presence of the insect throughout the season. Together they proxy different dimensions of the temperature regime affecting the pest, including the continuous period whereby the temperature remains above the minimum basal threshold required for the survival and continued development of the insect. Another set of temperature variables—specifically BIO 11, BIO 9, and BIO 1—are associated with limits that help delineate areas where fall armyworm could sustain a presence during the warmer seasons in higher and lower latitudes. The other important variables are related with solar radiation, notably BIO 26, BIO 21, BIO 24, BIO 23, BIO 20 and BIO 22. Almost all the radiation-based variables are associated with limits that delineate conditions considered extreme for the pest. The exception is variable BIO 23, which represents a measure of seasonality of solar radiation. Notably, the minimum temperature of the coldest week (BIO 6) is the most important contributing factor for the first principal component (PC1), while the lowest weekly radiation variable (BIO 22) was the most important for the second principal component (PC2).

**Figure 1:**
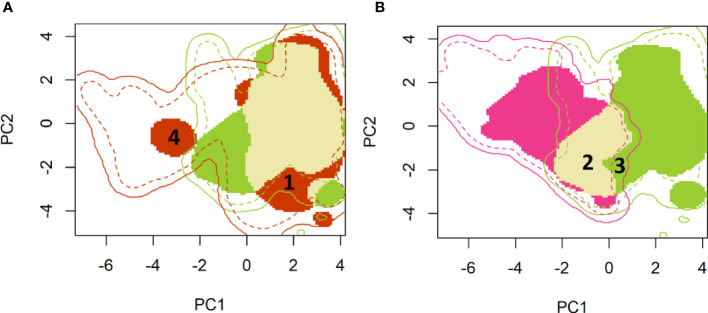
A principal component mapping of environmental niches for fall armyworm. **(A)**: Persistent native (green) vs newly invaded ranges (red) **(B)**: Persistent (green) versus historical seasonally invaded (pink) ranges. **(A)** Shows the persistent native environment – shown in green (mostly found in Latin America and Central America) versus the newly invaded ranges – shown in red (environment in the rest of the world). The light mustard color shows overlap between the persistent native and invaded ranges. Much of the invaded range reflects environmental conditions similar to those found in the native range, for example the area marked as “1” in the invaded range is shown tightly within the boundaries of the native environmental niche. A small portion of the newly invaded environment registers new environmental footprint relative to fall armyworm’s native range, e.g. the red colored area labeled “4” which is clearly outside the environemtnts availabe in the native range. **(B)** compares the environmetal niche of the persistent native populations -shown in green (Latin and Central America) versus the historically seasonally invaded-shown in Pink (North America). There is also an overlap in niche in these two populations shown in light mustard. High niche stability is noted in the historically seasonally invaded range where two-thirds of the invaded historical range has a similar environment to the native range (See area labeled 2, shown as overlap). The portion labeled ‘3’ shows that there are areas that fall armyworm has not reached despite having a similar native range niche. This niche unfilling could be due to land use, physical barriers etc.

A two-dimensional density representation of the fall armyworm occurrence data was then mapped on the environmental space represented by the first two principal components of the PCA analysis (**Figure 1**). Reported occurrences of fall armyworm that lie within the native range where the pest is likely to survive year-round (i.e., much of South America, Central America and southern locations of Texas and Florida) are represented by the green shaded area in Panel A and B. In Panel A, the red shaded area indicates the PC 1 and PC 2 representations of the climatology where fall armyworm occurrences occurred in the newly invaded areas in sub-Saharan Africa, India, China, and Australia. The climatologies where occurrences in both the persistent native and newly invaded areas overlap are indicated by the fawn color in Panel A.

As we quantify in **Table 4**, only a small portion of the newly invaded environment (i.e., expansion = 0.063, range [0, 1]) exhibits a new environmental footprint relative to fall armyworm’s native range, while the rest of the niche in the invaded range is shown to be similar to the environments available in the native range (see, for example, the red colored area labeled “1” in **Figure 1A** which falls within the bounds of the native range). This finding conforms with the results reported by Early et al. (40). In Panel B, the historically invaded range where the pest only survives seasonally (i.e., in North America) is indicated by the pink area, where the niche analysis gives a stability value of 0.66 (Range 1, 0), meaning that two-thirds of the invaded historical range has a similar environment to the native range (See area labeled 2 in **Figure 1B**). Notably, the niche analysis indicates a small environmental footprint (unfilling = 0.101, **Table 4**) where the pest is found in the native range but has yet to occupy a similar climatology in the historically invaded seasonal range in North America (see the sliver of green patch labeled 3 in **Figure 1B**). This could indicate either a lack of suitable host, or a barrier to dispersal from the native range or other seasonally invaded locations.

**Table 4:** Environmental overlap of various fall armyworm geographies.

Environmental niche category comparisons	Expansion	Stability	Unfilling
Native range *vs*. newly invaded range	0.063	0.937	0.393
Native range *vs*. historical seasonally invaded range	0.343	0.657	0.101

Niche Expansion is the proportion of the niche invaded by the species that is not occupied in its native range. Niche Stability is the proportion of the invaded environmental niche that concords with the occupied niche in the native range. Niche Unfilling is the proportion of the environment that is occupied in the native range but not occupied in the invaded range.

**Figure 2:**
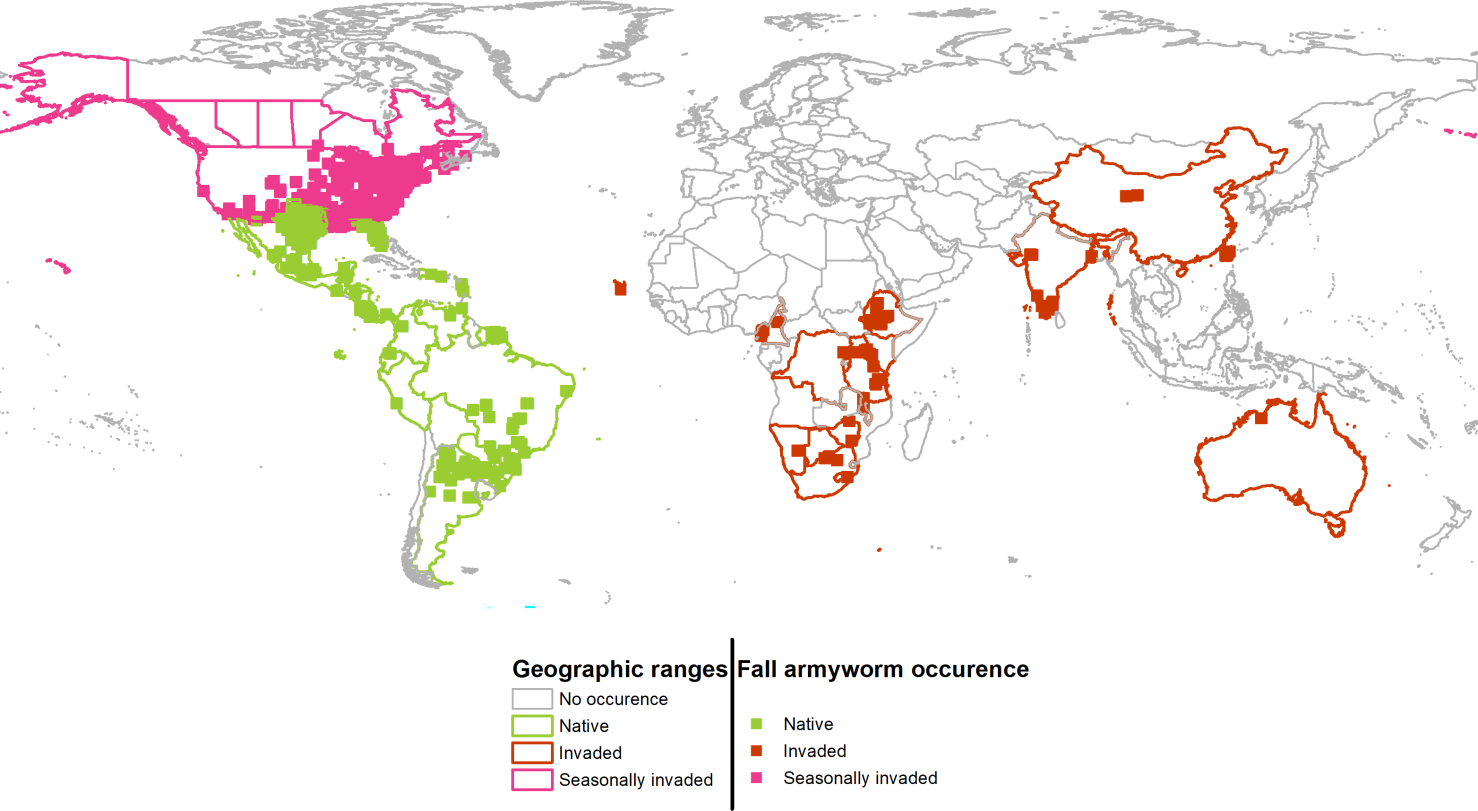
Geographic projections of the persistent (native) and seasonally invaded plus newly invaded ranges of fall armyworm. Colored boundaries indicate countries that contain FAW occurrences used for the environmental niche analysis.

Taking the plotted evidence in **Figure 1** as a whole, reveals that much of the environment under the persistent native range aligns with the pest’s newly invaded range. Thus, while the pest is spreading geographically, it is not spreading much beyond the environmental conditions that have long sustained it year-round throughout its persistent native range (Panel A) (see also 40). In both Panels, the solid lines indicate the limits of the environment available for each of the identified ranges. For example, the native range is taken to encompass an area that includes the countries reporting occurrence throughout Latin America, southern Texas and Florida. Thus, the solid green line in **Figure 1** indicates environments available throughout the countries with correspondingly green colored borders in **Figure 2**. The broken lines indicate the quantile (25%) of the environmental density used to delimit marginal climates. While country borders do not necessarily indicate a dispersal barrier for invading fall armyworm—especially for those countries that lack effective biosecurity measures to control cross-border invasions—they do provide some indication of the geographic extent to which an invading population might spread if the environmental conditions permitted (see, e.g., 67). Notably, the red circle labeled “4” in **Figure 1A** is located outside the solid green line that delineates the limits of the climatology available in the persistent native range, and suggests the pest has spread to some isolated environmental conditions in the newly invaded areas that lie outside the environment that prevails in the native range. However, since we are looking at the potential climatic suitability instead of the realized distribution, we did not use splines that represent such physical features to identify and remove some of the available environment in which FAW may have been excluded due to these physical features.

Cross referencing the plotted information in **Figures 1A, B** is informative. The overlap between the persistent native range in Latin America, and the historically invaded range in North America (see Panel A) reveals that the environmental conditions predicted to be outside of the persistent native range align with the seasonally invaded locations in North America shown in pink in Panel B. This suggests that some of the newly invaded areas are only seasonally affected and are likely being continuously re-invaded from adjacent areas where the species can establish a persistent population. Such seasonally invaded locations could be areas in South Africa and mainland China where our habitat suitability model (see below) has high GI values (i.e., climate only seasonally suitable) but not EI values (i.e., climate suitable for a persistent population.)

### 3.2 Climate suitability

Notably, almost all (i.e., 808 or 99.9%) of our spatially unique occurrence points fell within locales where we estimated that the pest can potentially complete at least one growth cycle (i.e., GI > 0 and no. of generations >= 1). In addition, 50.7% of the observations also fell within the EI > 0 range where the pest may persist year-to-year. A large majority (i.e., 389 out of 399; 97.5%) of the occurrence records that are not contained within EI-suitable locales were from North America, where the pest can only survive seasonally. That leaves just 10 (1.2%) of the occurrence locations with an EI = 0 prediction. On further investigation, 8 of these locations were in the temperate areas of Qinghai Sheng in China along with Mendoza and Cordoba in Argentina. Considering each of these locations have temperate climates, they are likely areas where fall armyworm can only occur seasonally. The remaining two occurrences that fell within EI = 0 locales were from central Mexico, where the occurrences overlapped with unsuitable climatic patches according to our CLIMEX suitability prediction.

**Figure 3:**
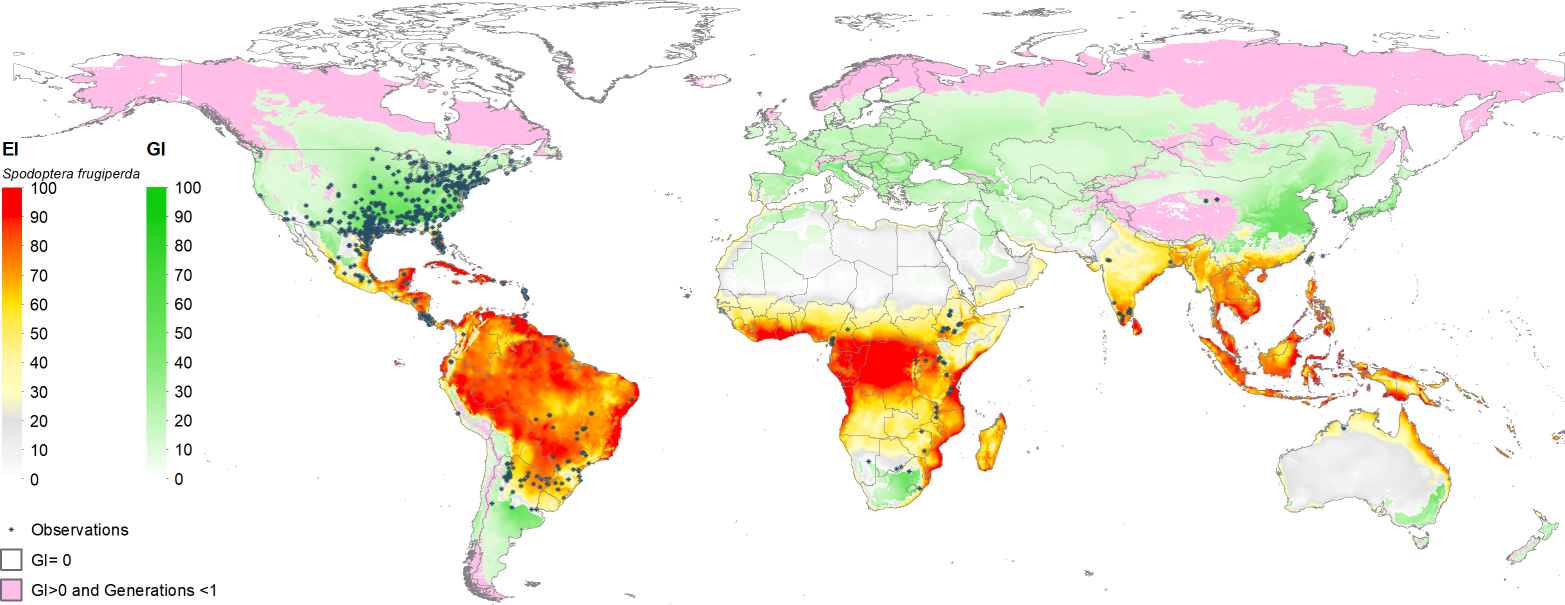
Worldwide seasonal and year-round risk from fall armyworm. *Observation in the legend indicates recorded geographic locations of fall armyworm used in this study.

**Figure 3** shows the estimated climate suitability of fall armyworm worldwide (i.e., spanning the world’s entire land mass). The green shaded locations represent areas where fall armyworm may propagate for at least one generation (GI > 0). These are typically the temperate areas in North America, Southern Argentina, South Africa and north eastern China, where the pest is unlikely to overwinter but may invade and undermine crop productivity during the growing season. The red-to-yellow shaded areas indicate locations where the pest can persist year-round (EI > 0). A transition in shading from light yellow to darker red represents increasing values of EI (that range from 0 to 100 by construction), indicative of ever-more climate suitable areas for year-round propagation of the pest. Clearly the warmer, moister areas of the world that lay within the tropics are where the pest is most likely to thrive throughout the year, and thus be especially problematic for multi-cropped areas where maize is often grown in both the rainy and dry seasons within a given calendar year. It also indicates areas where the insect is likely to establish itself following an invasion of this particularly mobile pest, thus increasing the odds of reoccurring crop losses. These EI > 0, GI > 0 areas are intrinsically more risky than areas (EI = 0, GI >0) where the pest does not overwinter but can propagate for at least one generation during a season once it finds its footing.

**Figure 4:**
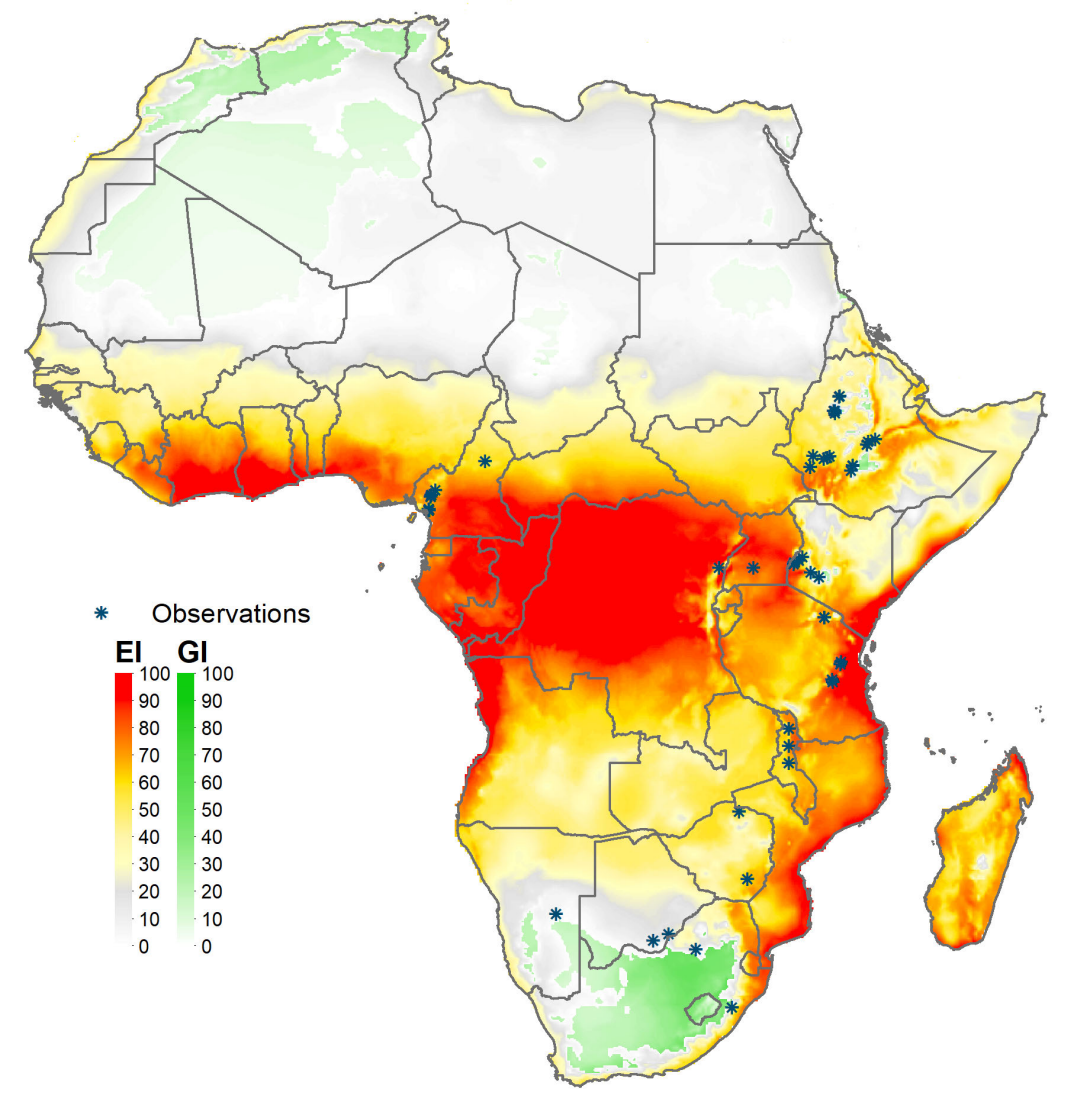
Climate suitability of fall armyworm in Africa. *Observation in the legend indicates recorded geographic locations of fall armyworm used in this study.

The global climate suitability projections based on our updated CLIMEX model, for the most part concord with those made by du Plessis et al. (9), but with some notable differences. The bulk of the world’s maize crop is grown in northerly latitudes–55% of maize growing locations and 75% of global maize output occur north of the Tropic of Cancer—where both du Plessis et al. (9) and our results indicate the potential for seasonal growth but not year-round survivability of the pest. Key differences occur in the northern and southern areas around the world that represent the transition zones between persistence and seasonality of the pest. More specifically, and in contrast with du Plessis et al. (9), we find that a) much of Mexico and more extensive areas throughout Florida and the southern border of Texas are suitable for permanent fall armyworm establishment, consistent with the now frequent incursion of fall armyworm in northern U.S. states reported by Nagoshi et al. (68). African countries such as Angola, Botswana, Namibia, and limited locations in the eastern coast of South Africa also support the pest year-round (in line with recent findings of 43), and c) the higher latitude region of India and areas in southeast China support persistent rather than seasonal occurrences of the pest. Our latter finding concords with the recent field survey and experimental evidence of (47, **Table 1** and **Figure 1**) who reported the ability of fall armyworm to overwinter in latitudes as high as 29.7° N in the subtropical regions of Xiuning, Huangshan and Anihui in China. **Figure 4** shows our modeled assessment of the climate suitability of fall armyworm focused on the African continent.

### 3.3 Maize risk

**Figure 5:**
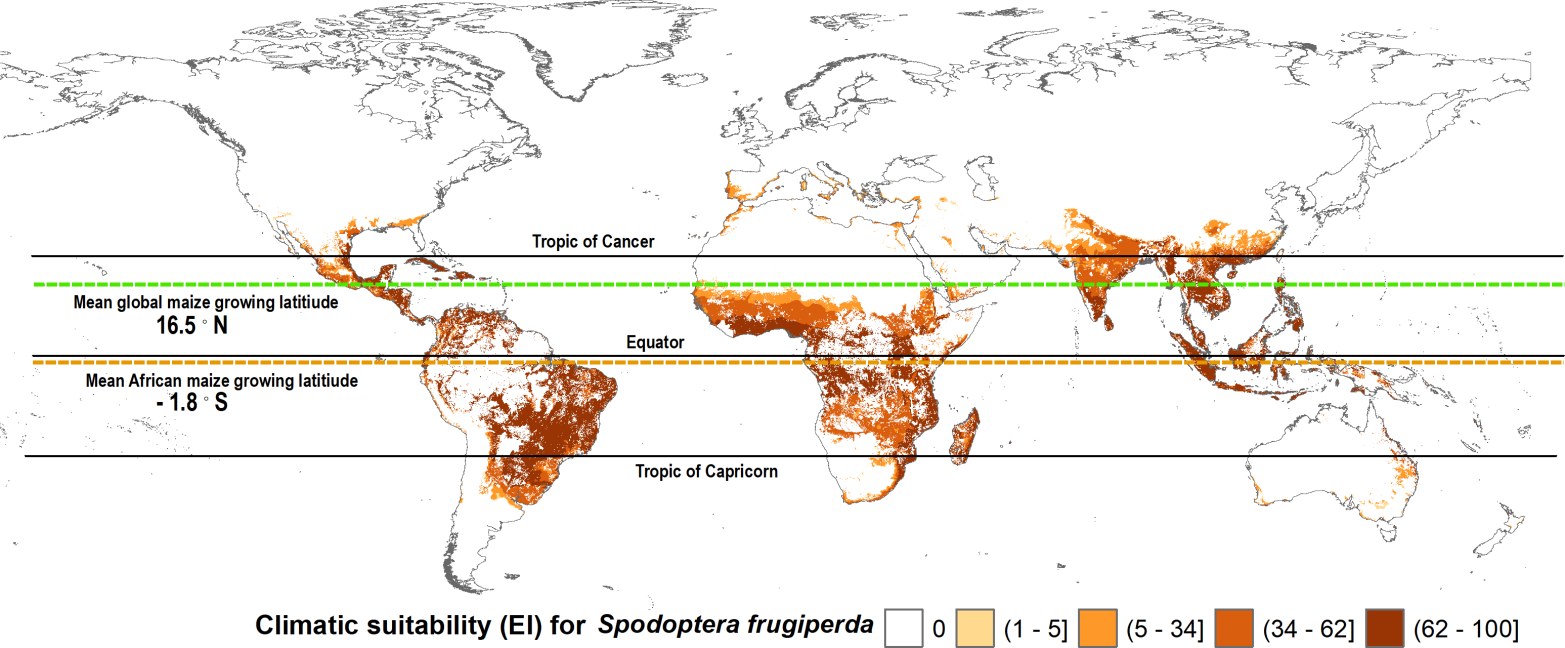
Fall armyworm risk for maize farmers worldwide. The spatial crop production data from You et al. (62) dataset was used to determine the maize extent of fall armyworm suitability.

Maize is a widely consumed staple food (and feed) crop throughout Africa. It is thus grown extensively, but not everywhere on the continent. **Figures 3**, **4** mapped the climate suitability of fall armyworm for the entire global and African landmasses, respectively. **Figure 5** shows the risk profile of the pest within the estimated spatial extent of maize production. For readability, we grouped the EI values into five risk categories: high risk areas having EI values between 62 and 100; upper-middle, 34 to 62; lower-middle,5 to 34; low, less than 5; and EI = 0 for no risk areas, the darker the shading the higher the risk. The figure also includes the mean maize growing latitude for Africa versus the mean maize growing latitude for the crop worldwide wide. Notably, the African mean growing latitude lies almost on the equator, indicating most, but by no means all, of the region’s maize is grown in tropical areas that are climate friendly for fall armyworm. A regional exception is South Africa that accounted for 15.2% of sub-Saharan Africa’s 2019 maize production, all of which takes place in temperate areas (see also 69, 70). The region’s following four top ranked producers—Nigeria (accounting for 14.8% of 2019 production), Ethiopia (13%), Tanzania (7.6%) and Kenya (5.2%)—are all located within tropical latitudes (70). In contrast, the worldwide mean latitude for maize lies well to the north of the equator, indicating a significant share of the crop is grown in more temperate climes—including the United States and China, together accounting for over half the world’s maize production in 2019—where fall armyworm is a more erratic and seasonal, rather than persistent, feature of the pest risk landscape facing maize producers.

**Figure 6:**
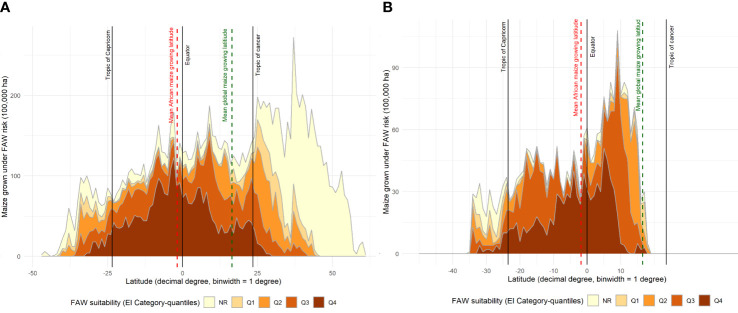
Maize production area under different categories of fall armyworm risk by latitude. **(A)**: Global maize production area shown by different fall armyworm risk categories. **(B)**: sub-Saharan Africa maize production area shown by different fall armyworm risk categories. Risk categories Q1 – Q4, indicated by categorizing the fall armyworm CLIMEX EI values into quantiles, Q1 for EI < 5; Q2 for EI = 5-34; Q3 for EI = 34-62; and Q4 for EI > 62.

An alternative, geo-located perspective of the risk that fall armyworm poses for maize producers around the world is given in **Figure 6**; an adaptation of the plotting procedure used by (71, **Figure 5**). In Panel A, we plot the share of the world’s maize production (by area) that occurs in one arc degree latitudinal bands arrayed left to right along the X axis from the most southerly production locale (46.6° south) in New Zealand to the most northerly point of production at 61.4° north in Russia. The area shares are then parsed into relative risk quintiles according to their respective EI values.

Around 76% of the world’s maize production occurs on land located north of the equator, with the bulk, 56% of the world’s total maize area located in the temperate north. Notably, significantly less of the world’s maize production occurs in tropical locales than crop production in total. Joglekar et al. (71)'s report that just under one-third (32.2%) of the total global value of crop production takes place in the tropics, compared with an estimated 18% of the world’s maize production (and 37% of maize area)—split roughly evenly north (10% of total tropical production) and south (8.5% of total tropical production) of the equator. Joglekar et al. (71)’s analysis of the global geography of crop production includes the circa 2005 production by value of the world’s 42 principal food, feed and fiber crops. Zeroing in on sub-Saharan Africa, 89% of the region’s total maize area (78% of its production) occurs in the tropics and most of the production occurs south of the equator, driven by South Africa, Tanzania and Malawi, the first, fourth and seventh ranked producers of maize in the region. In stark contrast, 71.4% of the region’s total crop production occurs north of the equator (71), propelled by Nigeria and Ethiopia who account for large shares of the region’s total crop production, respectively.

**Figure 6A** makes clear that the preponderance (60.3%) of the world’s *tropical* maize grows in climates that are at high risk from outbreaks of fall armyworm. Just 3.6% of the maize produced in *temperate* areas is at high risk, with the bulk being in the low-middle (6%) and low or no risk categories (83%). By contrast, the sub-Saharan Africa data plotted in Panel B shows that 42% of the region’s maize area falls within the highest risk category. Only 8.5% of the region’s maize area is in a low or no-risk category, with most of that area located in South(ern) Africa.

### 3.4 A multi-peril risk perspective of fall armyworm

**Figure 7:**
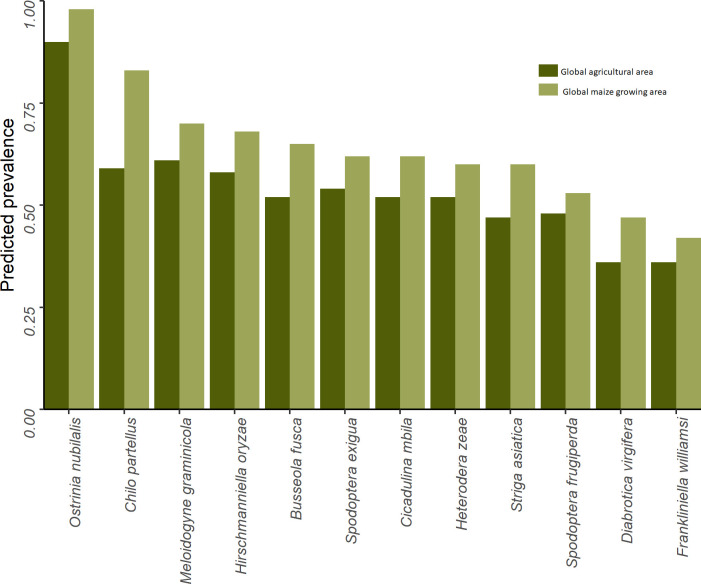
Global share of crop and maize area at risk from selected biotic threats.

**Figure 7** gives a high-level sense of the risk exposure for cropping agriculture worldwide to each of the 12 pests. The dark green bars indicate the share of the world’s total cropland at risk (i.e., EI > 0) for each of the pests; the lighter green bars indicate the respective maize areas at risk. The pests are arranged in rank order from left to right according to the global share of maize area at risk. We find that the climate conditions that affect crop production worldwide are likely to support at least one generation of *Ostrinia nubilalis* (European corn borer) annually on 90% of the world’s cropped area (and 98% of the maize area), making it the most geographically extensive pest of risk in this grouping. *Chilo partellus* (spotted stem borer) ranks the second most pervasive pest, putting 83% of the word’s maize area at risk. Regarding armyworms, 62% of the world’s maize area is deemed at risk from beet armyworm (*Spodoptera exigua*), with fall armyworm (*Spodoptera frugiperda)* putting 53% of global maize area at risk. Among this group of pests, *Frankliniella williamsi* (corn thrips) has the smallest risk footprint, but nonetheless still puts 42% of the world’s maize area at risk.

To gain a more nuanced sense of the multi-peril nature of the risk exposure, we quantified the co-location of the climate suitability of each of the twelve pests in our portfolio. To do so, maize pixels with *GI_p_* = *EI_p_* = 0 values for pest *p* were designated climate-unsuitable for that particular pest (and assigned a “habitation” value of 0); those with values other than zero were deemed climate-suitable and assigned habitation values of 1. Our co-habitation measure is a simple sum of the pest-specific habitation values for each pixel. We then spatially intersected these co-habitation values against pixilated estimates of the area growing maize to estimate the share of the world’s maize crop by region that is at risk from the crop loss consequences of multiple pests. **Table 5** summarizes the results from this analysis.

**Table 5:** Fall armyworm from a multi-peril risk perspective.

Region	Maize Growing Area		FAW Risk		Conditional Pest Co-Suitability											
			(GI >0)	(EI >0)	0	1	2	3	4	5	6	7	8	9	10	11
*Column Number*	(1)	(2)	(3)	(4)	(5)	(6)	(7)	(8)	(9)	(10)	(11)	(12)	(13)	(14)	(15)	(16)
*Units*	*(ha, million)*	(%)	(%)	(%)	(%)	(%)	(%)	(%)	(%)	(%)	(%)	(%)	(%)	(%)	(%)	(%)
Asia and Pacific	44.6	29.5	99.3	43.4	1.4	1.6	6.4	28.2	7.6	3.4	4.8	4.4	2.8	15.3	20.2	3.8
Europe and Central Asia	12.1	8.0	99.7	2.8	0.3	1.2	21.2	33.5	10.2	13.2	10.9	5.1	1.2	2.3	0.9	0.1
High-Income	37.8	25.0	99.9	2.3	0.3	0.1	4.8	36.9	35.9	4.7	1.9	4.7	4.4	2	3.2	1
Latin America and Caribbean	27.4	18.1	99.7	90.7	0.5	0.1	0.1	0.3	0.4	0.6	0.9	1.3	1.3	25.7	61.8	6.9
Middle East and Northern Africa	1.6	1.1	99.9	79.3	47.1	15.1	5.6	4.4	4.6	4.9	3.1	8.5	4	2.4	0.4	0
Sub-Saharan Africa	27.7	18.3	99.9	91.8	0	0.1	0	0.3	0.5	0.8	1.6	2	3.8	52.5	38.1	0.2
Total	151.3	100.0	99.7	47.7	1.1	0.8	4.9	20.4	12.2	3.6	3.2	3.6	3	19.5	25	2.7

Conditional pest co-suitability indicates the share of maize area in the respective region that is deemed climate suitable for up to 11 pests (**Table 2**), conditional on that maize area also being designated as climate suitable for fall armyworm. The numbers in brackets in the table are column numbers assigned to make it easy to call out specific figure in the text.

Given that almost all the world’s maize crop is susceptible to a seasonal infestation of FAW (GI > 0) based on our climate suitability model (see **Figure 3** and **Table 5**, Column 3), most of the world’s maize crop is susceptible to a seasonal infestation of fall armyworm. However, there are stark geographical differences in the year-round risk exposure to the pest (EI > 0, as in column 4). Less than 3% of the maize area in High-income and Europe and Central Asian countries have climates that are suitable for persistent exposure to the pest; both temperate regions of the world that account for 65.2 million hectares of maize (33% of the world total). In contrast, more than 90% of the maize growing areas in Latin America and the Caribbean (LAC) and sub-Saharan Africa (SSA)—together accounting for 36% of the world’s maize area—are climate suitable for year-round survivability of the pest. This places farmers in these parts of the world at much high risk of crop losses from fall armyworm given that once the pest invades it has a higher odds of persisting and re-infecting crops from year-to-year.

Columns 5 to 16 are *conditional* area shares. They indicate the share of maize area with year-round susceptibility to the presence of fall armyworm that has *additional* risk exposure to the other 11 pests listed in **Table 5**. Thus, looking across the “Total” row, one-fifth (20.4%) of the world’s maize area with a climate that may sustain year-round growth of fall armyworm worldwide can also sustain a persistent presence of three other maize pests. Another fifth of the total maize area that is fall armyworm friendly can sustain a further nine pests and, markedly, one-quarter of the area can sustain ten more pests.

**Table 5** also reveals dramatic geographical differences in the multi-peril risk exposure of maize farmers that are also susceptible to crop damage from fall armyworm. For example, a minor share (2.3%) of the maize area in High-income countries has year-round susceptibility to fall armyworm, while 72.8% of that area is climate suitable for another 3 or 4 pests. Maize producers in Europe and Central Asia face a similar multi-peril risk profile. However, the pest risk realities for farmers in Latin America are dramatically different. Most (90.7%) of the maize area in this part of world is grown in climates that result in year-round risk from fall armyworm. Moreover, a large share of this acreage (61.8%) is also susceptible to infection year-round from an additional 10 pests. Maize farmers in sub-Saharan Africa face even higher multiperil risk exposures. An estimated 91.8% of this maize area in sub-Saharan Africa can support fall armyworm year-round, and over half (52.5%) of that area can sustain growth of a further nine pests; 38.1% of the fall-armyworm susceptible maize area can support an additional ten pests.

**Figure 8:**
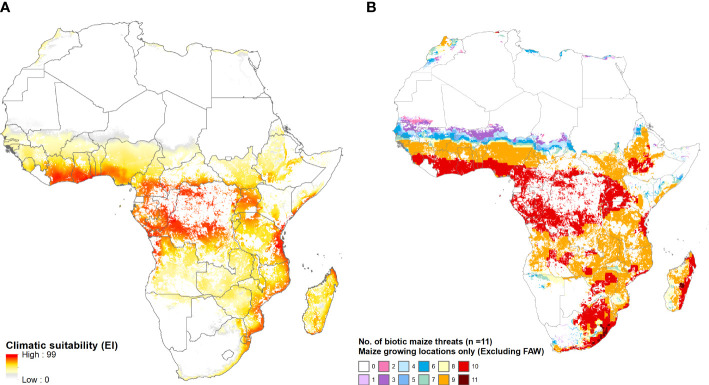
The African geography of multi-peril pest risk. **(A)**: Fall armyworm climate suitability. **(B)**: Co-habitation counts for 11 maize pests. Panel **(A)** plots climatic suitability (EI values) of fall armyworm within the entire maize growing area throughout Africa. Panel **(B)** plots the locations deemed suitable for up to 11 biotic threats (**Table 2**), within the maize producing area in the region.

**Figure 8** provides a mapped, spatially explicit representation of the multi-peril risks for Africa that are summarized in **Table 5**. Panel A maps the year-round vulnerability of the maize area in Africa to fall armyworm. Panel B shows the pattern of co-habitation (i.e., climate co-suitability) for the 11 additional pests listed in **Table 2**. Like the risk from fall armyworm, the multiperil risk exposure for maize producers in Africa is pervasive and extreme. Most (specifically 95% or more, and in many instances 99 or 100%) of the maize area in most African countries supports a year-round presence of the pest. In addition, most of these areas have climates that also provide growing conditions that support a persistent presence of nine or ten of the pests listed in **Table 2**. The standout exceptions are South Africa and Lesotho, the latter country located entirely within South African borders and geographically adjacent to the maize triangle where a majority of South African maize is produced. While the maize climate for both these countries is suitable for seasonal growth of fall armyworm, little of the cropped land in both countries will likely support the pest year-round. Nonetheless, much (59% of Lesotho and 78% for South Africa) of the small area at higher risk from fall armyworm is also climate suitable for ten of the pests in our evaluation frame.

## 4 Discussion

In general, climates that favor maize production are also seasonally suitable for fall armyworm infestations. Around half the world’s maize area, mostly in the moist and warm tropical locales, is also likely to sustain the development of the pest year-round. With the exception of South Africa, the leading producer of maize on the continent (and Lesotho a comparatively small producer), Africa is favorable climatically for FAW. Moreover, most of the region’s maize area has a climate that is also hospitable to nine or ten other pests that prove problematic for maize producers (**Table 5**). This means the multi-peril risk exposure is especially worrisome for many of the maize producers throughout Africa, many of whom are smallholders with a heavy reliance on maize as a source of household income or for direct consumption by the farm households who grow the crop.

Crop losses associated with particular pests have the same livelihood and well-being implications for farm households irrespective of the pest in questions. Thus, it follows that meaningful strategies to deal with fall armyworm, or any other crop pest are best conceived and executed from a multi-peril pest perspective, rather than a piece-meal, pest-by-pest approach. However, in dealing with crop pests, tradeoffs are inevitable, and so evidence on the multi-peril nature of the risk is key to making cost-effective strategic decisions on what combination of pest management strategies (be they genetic, chemical or crop management) to target for development and deployment. The spatially-explicit, co-habitation metrics developed here are a first step at informing these choices. Taking additional steps to prioritize action in a multi-peril setting requires significant additional data and analytical efforts.

The evidence we present here indicates that European corn borer (*Ostrinia nubilalis)* poses the most geographically extensive pest risk for maize, spanning 98% of the world’s maize cropping area (see **Figure 7**). This pest is also potentially problematic throughout sub-Saharan Africa, but not to the same degree. Likewise, while western corn rootworm (*Diabrotica virgifera virgifera*) is especially problematic in the temperate areas that predominate maize production in North America, a comparatively small share (9.8%) of the maize growing area in Africa is climate-suitable for this pest. These differential multi-peril risk profiles suggest that regional priorities may not necessarily align with global priorities, thus increasing the value of this type of pest risk information to better align pest mitigation efforts overall in ways that optimize the use of scarce R&D and related resources.

Crop breeding for pest resistance is a proven strategy for dealing with the crop losses that come from pest outbreaks. While improved maize varieties are used throughout Africa, adoption is far from complete, the intensity of use varies markedly among countries, and in general lags many decades behind the extent of uptake in rich countries. Moreover, the rate of varietal turnover is comparatively low (72), meaning many of these varieties likely lack the resistance genes required to protect them from newly invaded pests such as FAW, or long established, but ever-evolving, pests that have skirted around earlier forms of genetic resistance. Pardey et al. (73) reveal there is limited use of chemicals throughout much of African agriculture, some of which controls crop pests. These thin agricultural input markets are in part a reflection of the difficulties (and costs) of servicing farmers who are mostly smallholders and often distant from markets. Joglekar et al. (71) reported that “… just 54.6 percent of the [African] continent’s [mainly rural] population can reach … small urban markets [with at least 20,000 inhabitants] in 3 hours or less…” The costs of moving goods and services to and from farms thus undermines off-farm market participation. This suggests that crop management and, especially, genetic solutions to addressing the multi-peril pest problems of African farmers may be more cost-effective than chemical solutions, which require more frequent and timely trips to markets to secure the necessary pest-mitigating inputs (fungicides, insecticides) as seasonal pest infections unfold. While integrated pest management (IPM) practices constitute another, often complementary, strategy for controlling crop pests, and perhaps especially so in tropical regions where natural enemies can have year-round survivability (74), IPM is likewise not widely adopted throughout the developing world (75).

Changing climate poses yet another set of challenges in targeting and implementing pest control practices. Our multi-peril assessment is based on a 30-year (1961-1990) average climatology for each pixel on the planet. In principle, the same modeling and multiperil assessment procedures can be conducted using projected climates, as Ramirez-Cabral et al. (39) did for FAW using two general circulation models (GCMs) and two carbon emission scenarios. Notably, Liu et al. (76) used a similar approach that considered both climate and land use changes and reported that the projected geographic distribution of FAW was shaped more by changes in land use than climate. With any such models, there is always the problem of which global climate projection to use, and the accuracy of those projections (77), especially at the more refined spatial resolutions required for meaningful and operationally relevant pest risk assessments. Notwithstanding these complications, the spatially explicit, multi-peril pest risk approach we present here can be used to both benchmark future multi-peril pest risk assessments under prospective changes in climate, while also informing current and nearer-term strategies to target market and government resources in ways that have the most beneficial effect in mitigating the complex of crop pests that pose the most risk for farmers growing particular crops in specific locales.

## Data availability statement

The original contributions presented in the study are included in the article/**Supplementary Material**. Further inquiries can be directed to the corresponding authors.

## Author contributions

SS, PP, YC, LD and RD conceived, brainstormed, and designed the idea for this article. SS performed the analysis, SS and PP analysed the literature, wrote the manuscript, and took part in improving the manuscript. All authors reviewed the manuscript. All authors contributed to the article and approved the submitted version.

## Footnotes

1 In some modeling contexts, where both presences and absences of a species are used for climatic suitability projection, observations of no occurrence (i.e., where the pest is scouted but not found) have modeling value in addition to occurrence data (i.e., where the pest is found).

## References

[1] Goergen G Kumar PL Sankung SB Togola A Tamò M. First report of outbreaks of the fall armyworm spodoptera frugiperda (JE Smith)(Lepidoptera, noctuidae), a new alien invasive pest in West and central Africa. PloS One (2016) 11:e0165632. doi: 10.1371/journal.pone.0165632 27788251 PMC5082806

[2] Abrahams P Bateman M Beale T Clottey V Cock M Colmenarez Y. Fall armyworm: impacts and implications for Africa. Nairobi, Kenya: Evidence note (2)Center for Agriculture and Bioscience International-CABI (2017).

[3] Day R Abrahams P Bateman M Beale T Clottey V Cock M. Fall armyworm: impacts and implications for Africa. Outlooks Pest Manage (2017) 28:196–201. doi: 10.1564/v28_oct_02

[4] Prasanna B Bruce A Winter S Otim M Asea G Sevgan S. Host plant resistance to fall armyworm. In: Fall armyworm in Africa: a guide for integrated pest management. Mexico: USAID; CIMMYT (2018) 45–62.

[5] Sisay B Tefera T Wakgari M Ayalew G Mendesil E. The efficacy of selected synthetic insecticides and botanicals against fall armyworm, spodoptera frugiperda, in maize. Insects (2019) 10:45. doi: 10.3390/insects10020045 30717302 PMC6410260

[6] CIMMYT. New global research alliance joins fight against fall armyworm. El Batan: International consortium established to connect research with practical field solutions against pest (2018).

[7] USAID. USAID announces winners of the feed the future fall armyworm tech prize.in USAID. Washington DC: Foundation for Food & Agriculture Research (FFAR) (2018). Available at: https://foundationfar.org/news/usaid-announces-winners-of-the-feed-the-future-fall-armyworm-tech-prize/

[8] USAID. USAID combats fall armyworm infestation in the democratic republic of the congo.in USAID. Washington DC: U.S. Embassy Kinshasa. (2019). Available at: https://cd.usembassy.gov/usaid-combats-fall-armyworm-infestation-in-the-democratic-republic-of-the-congo.

[9] du Plessis H Van den Berg J Ota N Kriticos D. Spodoptera frugiperda. fall armyworm. Canberra, Australia: CLIMEX modelling. CSIRO-InSTePP Pest Geography (2018).

[10] Pashley DP. Host-associated genetic differentiation in fall armyworm (Lepidoptera: Noctuidae): a sibling species complex? Ann Entomol Soc America (1986) 79:898–904. doi: 10.1093/aesa/79.6.898

[11] Pashley DP Hammond AM Hardy TN. Reproductive isolating mechanisms in fall armyworm host strains (Lepidoptera: Noctuidae). Ann Entomol Soc America (1992) 85:400–5. doi: 10.1093/aesa/85.4.400

[12] Nagoshi RN Koffi D Agboka K Tounou KA Banerjee R Jurat-Fuentes JL. Comparative molecular analyses of invasive fall armyworm in Togo reveal strong similarities to populations from the eastern united states and the greater antilles. PloS One (2017) 12:e0181982. doi: 10.1371/journal.pone.0181982 28738081 PMC5524310

[13] Otim MH Tay WT Walsh TK Kanyesigye D Adumo S Abongosi J. Detection of sister-species in invasive populations of the fall armyworm spodoptera frugiperda (Lepidoptera: Noctuidae) from Uganda. PloS One (2018) 13:e0194571. doi: 10.1371/journal.pone.0194571 29614067 PMC5882101

[14] Meagher R Nagoshi R Stuhl C Mitchell E. Larval development of fall armyworm (Lepidoptera: Noctuidae) on different cover crop plants. Florida Entomol (2004) 87(4):454–60. doi: 10.1653/0015-4040(2004)087[0454:LDOFAL]2.0.CO;2

[15] Nagoshi RN Silvie P Meagher RL Lopez J Machado V. Identification and comparison of fall armyworm (Lepidoptera: Noctuidae) host strains in Brazil, Texas, and Florida. Ann Entomol Soc America (2007) 100:394–402. doi: 10.1603/0013-8746(2007)100[394:IACOFA]2.0.CO;2

[16] Nagoshi RN Koffi D Agboka K Adjevi AKM Meagher RL Goergen G. The fall armyworm strain associated with most rice, millet, and pasture infestations in the Western hemisphere is rare or absent in Ghana and Togo. PloS One (2021) 16:e0253528. doi: 10.1371/journal.pone.0253528 34153077 PMC8216543

[17] Cock MJ Beseh PK Buddie AG Cafá G Crozier J. Molecular methods to detect spodoptera frugiperda in Ghana, and implications for monitoring the spread of invasive species in developing countries. Sci Rep (2017) 7:1–10. doi: 10.1038/s41598-017-04238-y 28642581 PMC5481405

[18] Tay WT Rane RV Padovan A Walsh TK Elfekih S Downes S. Global population genomic signature of spodoptera frugiperda (fall armyworm) supports complex introduction events across the old world. Commun Biol (2022) 5:1–15. doi: 10.1038/s42003-022-03230-1 35393491 PMC8989990

[19] Nagoshi RN Goergen G Koffi D Agboka K Adjevi AKM Du Plessis H. Genetic studies of fall armyworm indicate a new introduction into Africa and identify limits to its migratory behavior. Sci Rep (2022) 12:1–12. doi: 10.1038/s41598-022-05781-z 35121788 PMC8816908

[20] Luginbill P. The fall armyworm. Washington DC p: USDA Technical Bulletin (Vol. 34). United States Department of Agriculture (1928).

[21] Mitchell ER. USDA Technical bulletin no. 34: The legacy of Philip luginbill. Hollywood, Florida, USA: Florida Entomologist, 452-455 (1986).

[22] Pogue MG. A world revision of the genus spodoptera Guenée:(Lepidoptera: Noctuidae). Philadelphia, USA: American Entomological Society Philadelphia (2002).

[23] Pair S Raulston J Sparks A Westbrook J Douce G. Fall armyworm distribution and population dynamics in the southeastern states. Florida Entomologist (1986) p. 468–87.

[24] Westbrook J Nagoshi R Meagher R Fleischer S Jairam S. Modeling seasonal migration of fall armyworm moths. Int J biometeorol (2016) 60:255–67. doi: 10.1007/s00484-015-1022-x 26045330

[25] Sparks AN. A review of the biology of the fall armyworm. Florida Entomologist (1979) p. 82–7.

[26] Brown E Dewhurst C. The genus spodoptera (Lepidoptera, noctuidae) in Africa and the near east. Bull entomological Res (1975) 65:221–62. doi: 10.1017/S0007485300005939

[27] Haggis MJ. Distribution of the African armyworm, spodoptera exempta (Walker)(Lepidoptera: Noctuidae), and the frequency of larval outbreaks in Africa and Arabia. Bull entomological Res (1986) 76:151–70. doi: 10.1017/S0007485300015376

[28] Rose D Dewhurst C Page W. The African armyworm handbook: the status, biology, ecology, epidemiology and management of spodoptera exempta (Lepidoptera: Noctuidae). Natural Resour Institute Univ Greenwich (2000) 1. Available at: http://www.armyworm.org/wp-content/uploads/2018/08/TheAfricanArmywormHandbook_2014revision.pdf

[29] Pardey PG Beddow J Kriticos D Hurley T Park R Duveiller E. Right-sizing stem-rust research. Science (2013) 340:147–8. doi: 10.1126/science.122970 23580514

[30] Koo J Pardey PG. HarvestChoice: Supporting strategic investment choices in agricultural technology development and adoption. Intl Food Policy Res Inst (2020) 1–12. doi: 10.2499/p15738coll2.133807

[31] Kocmánková E Trnka M Eitzinger J Formayer H Dubrovský M Semerádová D. Estimating the impact of climate change on the occurrence of selected pests in the central European region. Climate Res (2010) 44:95–105. doi: 10.3354/cr00905

[32] Zheng X-L Wang P Cheng W-J Wang X-P Lei C-L Heckel D. Projecting overwintering regions of the beet armyworm, spodoptera exigua in China using the CLIMEX model. J Insect Sci (2012) 12:1–13. doi: 10.1673/031.012.1301 PMC3467093 22934543

[33] Hauptfleisch K Yonow T Kriticos DJ Ota N. Busseola fusca (African stem borer). Saint Paul, Minnesota: InSTePP (HarvestChoice), University of Minnesota (2014). Available at: https://econpapers.repec.org/paper/agshcppgb/249748.htm

[34] Mylonas P Yonow T Kriticos DJ. Cicadulina mbila (Naudé) (Maize leafhopper). Saint Paul, Minnesota: InSTePP (HarvestChoice), University of Minnesota (2014). Available at: https://econpapers.repec.org/paper/agshcppgb/249747.htm

[35] Nail K Kriticos DJ Scott JK Yonow T Ota N. Striga asiatica (Witchweed). Saint Paul, Minnesota: InSTePP (HarvestChoice), University of Minnesota (2014). Available at: https://econpapers.repec.org/paper/agshcppgb/249748.htm

[36] Yonow T Kriticos DJ. Diabrotica virgifera virgifera. Saint Paul, Minnesota: Western Corn Rootworm (2014).

[37] Yonow T Kriticos DJ Ota N Van Den Berg J Hutchison WD. The potential global distribution of chilo partellus, including consideration of irrigation and cropping patterns. J Pest Sci (2017) 90:459–77. doi: 10.1007/s10340-016-0801-4 PMC5320010 28275325

[38] Singh SK Kriticos DJ Ota N Hodda M. Potential distribution and biosecurity risks from three economically important plant-parasitic nematodes. Ann Appl Biol (2022) 180:371–82. doi: 10.1111/aab.12739

[39] Ramirez-Cabral NYZ Kumar L Shabani F. Future climate scenarios project a decrease in the risk of fall armyworm outbreaks. J Agric Sci (2017) 155:1219–38. doi: 10.1017/S0021859617000314

[40] Early R González-Moreno P Murphy ST Day R. Forecasting the global extent of invasion of the cereal pest spodoptera frugiperda, the fall armyworm. NeoBiota (2018) 40:25–50. doi: 10.3897/neobiota.40.28165

[41] Baloch MN Fan J Haseeb M Zhang R. Mapping potential distribution of spodoptera frugiperda (Lepidoptera: Noctuidae) in central Asia. Insects (2020) 11:172. doi: 10.3390/insects11030172 32182795 PMC7142664

[42] Maino JL Schouten R Overton K Day R Ekesi S Bett B. Regional and seasonal activity predictions for fall armyworm in Australia. Curr Res Insect Sci (2021) 1:100010. doi: 10.1016/j.cris.2021.100010 36003595 PMC9387490

[43] Timilsena BP Niassy S Kimathi E Abdel-Rahman EM Seidl-Adams I Wamalwa M. Potential distribution of fall armyworm in Africa and beyond, considering climate change and irrigation patterns. Scientific Reports (2022) 12(539). doi: 10.21203/rs.3.rs-196606/v1 PMC8752590 35017586

[44] Sutherst RW Maywald G. A computerised system for matching climates in ecology. Agriculture Ecosyst Environ (1985) 13:281–99. doi: 10.1016/0167-8809(85)90016-7

[45] Kriticos DJ Maywald GF Yonow T Zurcher EJ Herrmann NI Sutherst R. Exploring the effects of climate on plants, animals and diseases. CLIMEX Version (2015) 4:184.

[46] Fan J Haseeb M Ren Q Tian T Zhang R Wu P. Factoring distribution and prevalence of fall armyworm in southwest China. J Appl Entomol (2021) 145:295–302. doi: 10.1111/jen.12852

[47] Yang X-m Song Y-f Sun X-x. Shen X-j Wu Q-l. Zhang H-w. Population occurrence of the fall armyworm, spodoptera frugiperda (Lepidoptera: Noctuidae), in the winter season of China. J Integr Agric (2021) 20:772–82. doi: 10.1016/S2095-3119(20)63292-0

[48] Osabutey AF Seo BY Kim A Ha TAT Jung J Goergen G. Identification of a fall armyworm (Spodoptera frugiperda)-specific gene and development of a rapid and sensitive loop-mediated isothermal amplification assay. Sci Rep (2022) 12:1–10. doi: 10.1038/s41598-022-04871-2 35042914 PMC8766445

[49] Valdez-Torres JB Soto-Landeros F Osuna-Enciso T Báez-Sañudo MA. Phenological prediction models for white corn (Zea mays l.) and fall armyworm (Spodoptera frugiperda JE smith). Agrociencia (2012) 46:399–410.

[50] Wu P Head ML Liu C Haseeb M Zhang R. The high invasion success of fall armyworm is related to life-history strategies across a range of stressful temperatures. Pest Manage Sci (2022) 78:2398–404. doi: 10.1002/ps.6867 35277917

[51] GBIF. GBIF occurrence download. Spodoptera frugiperda Smith & Abbot (2021). Available at: https://www.gbif.org/species/5109855

[52] ACAPS. Armyworm outbreak in Africa. Thematic Report. (2017). Available at: https://reliefweb.int/sites/reliefweb.int/files/resources/20170425_acaps_thematic_report_southern_africa_armyworms_update.pdf

[53] Johnson S. Migration and the life history strategy of the fall armyworm, spodoptera frugiperda in the western hemisphere. Int J Trop Insect Sci (1987) 8:543–9. doi: 10.1017/S1742758400022591

[54] Kriticos DJ Webber BL Leriche A Ota N Macadam I Bathols J. CliMond: global high-resolution historical and future scenario climate surfaces for bioclimatic modelling. Methods Ecol Evol (2012) 3:53–64. doi: 10.1111/j.2041-210X.2011.00134.x

[55] Hijmans RJ Cameron SE Parra JL Jones PG Jarvis A. Very high resolution interpolated climate surfaces for global land areas. Int J Climatol: A J R Meteorological Soc (2005) 25:1965–78. doi: 10.1002/joc.1276

[56] Fick SE Hijmans RJ WorldClim 2: new 1km spatial resolution climate surfaces for global land areas. International Journal of Climatology (2017) 7 (12) ::4302–15.

[57] Breiman L. Random forests. Mach Learn (2001) 45:5–32. doi: 10.1023/A:1010933404324

[58] R Core Team. A language and environment for statistical computing. Vienna, Austria: R Foundation for Statistical Computing (2021).

[59] Liaw A Wiener M. Classification and regression by randomForest (2002). Available at: http://cran.r-project.org/web/packages/randomForest/index.html (Accessed 2012 Jun 13).

[60] Di Cola V Broennimann O Petitpierre B Breiner FT d'Amen M Randin C. Ecospat: an r package to support spatial analyses and modeling of species niches and distributions. Ecography (2017) 40:774–87. doi: 10.1111/ecog.02671

[61] ESRI. ArcMap. Redlands, CA (2021) Environmental Systems Research Institute (ESRI). Available at: https://www.esri.com/

[62] You L Wood S Wood-Sichra U Wu W. Generating global crop distribution maps: From census to grid. Agric Syst (2014) 127:53–60. doi: 10.1016/j.agsy.2014.01.002

[63] Beddow JM Hurley TM Kriticos DJ Pardey PG. Measuring the global occurrence and probabilistic consequences of wheat stem rust. Harvest Choice Tech Note (2013) 340(6129):147–8.. doi: 10.1126/science.122970 23580514

[64] Garcia A Godoy W Thomas J Nagoshi R Meagher R. Delimiting strategic zones for the development of fall armyworm (Lepidoptera: Noctuidae) on corn in the state of Florida. J economic entomology (2018) 111:120–6. doi: 10.1093/jee/tox329 29267899

[65] Richardson CW. Dependence structure of daily temperature and solar radiation. Trans ASAE (1982) 25:735–0739. doi: 10.13031/2013.33604

[66] Haynes KJ Tardif JC Parry D. Drought and surface-level solar radiation predict the severity of outbreaks of a widespread defoliating insect. Ecosphere (2018) 9:e02387. doi: 10.1002/ecs2.2387

[67] Senay SD Worner SP Ikeda T. Novel three-step pseudo-absence selection technique for improved species distribution modelling. PloS One (2013) 8:e71218. doi: 10.1371/journal.pone.0071218 23967167 PMC3742778

[68] Nagoshi RN Fleischer S Meagher RL. Texas Is the overwintering source of fall armyworm in central Pennsylvania: implications for migration into the northeastern united states. Environ entomology (2009) 38:1546–54. doi: 10.1603/022.038.0605 20021748

[69] Pardey PG Greyling JC. Measuring maize in south Africa: The shifting structure of production during the twentieth century 1904–2015. Agrekon (2019) 58:21–41. doi: 10.1080/03031853.2018.1523017

[70] FAO. FAOSTAT crop statistics database. Rome, Italy: FAO (2021). Available at: http://www.fao.org/faostat/en/#data/QC.

[71] Joglekar AB Pardey PG Wood Sichra U. “Where in the World are Crops Grown?” HarvestChoice Brief. St. Paul, MN: International Science & Technology Practice and Policy (InSTePP) Center, University of Minnesota and Washington, D.C.: International Food Policy Research Institute (IFPRI) (2016). Available at: https://econpapers.repec.org/paper/agshcppgb/249748.htm

[72] Atlin GN Cairns JE Das B. Rapid breeding and varietal replacement are critical to adaptation of cropping systems in the developing world to climate change. Global Food Secur (2017) 12:31–7. doi: 10.1016/j.gfs.2017.01.008 PMC5439485 28580238

[73] Pardey PG Chan-Kang C Liebenberg F Luby I Joglekar A Senay S. What do we know about (Procured) input use in African agriculture? , department of applied economics. Minnesota: University of Minnesota, St. Paul (2017).

[74] Hollingsworth RG. Insect pest management of tropical versus temperate crops; patterns of similarities and differences in approach. Int Soc Hortic Sci (ISHS) Leuven Belgium (2011) 894, 45–56. doi: 10.17660/ActaHortic.2011.894.3

[75] Alwang J Norton G Larochelle C. Obstacles to widespread diffusion of IPM in developing countries: lessons from the field. J Integrated Pest Manage (2019) 10:10. doi: 10.1093/jipm/pmz008

[76] Liu T Wang J Hu X Feng J. Land-use change drives present and future distributions of fall armyworm, spodoptera frugiperda (JE Smith)(Lepidoptera: Noctuidae). Sci Total Environ (2020) 706:135872. doi: 10.1016/j.scitotenv.2019.135872 31855628

[77] Beaumont LJ Hughes L Pitman AJ. Why is the choice of future climate scenarios for species distribution modelling important? Ecol Lett (2008) 11:1135–46. doi: 10.1111/j.1461-0248.2008.01231.x 18713269

