# Supplementary material for: Fall armyworm from a maize multi-peril pest risk perspective
Source: Front Insect Sci. 2022 Dec 19;2:971396. doi: 10.3389/finsc.2022.971396 (PMC10926406; doi:10.3389/finsc.2022.971396)
Supplement: Supplementary file 1 [file DataSheet_1.pdf]

Supplementary Material

Content

Supplementary Figure 1. The two variables that contributed most to the first and second principal components used to map *S. frugiperda* environmental niches.

Supplementary Table 1. Pool of bioclimatic variables available for fall armyworm environmental niche modeling prior to variable selection.

Supplementary Table 2. Geographical and other spatial attributes of observed occurrence data**.**

Supplementary Table 3: FAW CLIMEX parameters developed for the present study.

Supplementary Section 1: Comparison of Ramirez-Cabral et al. (2017), du Plessis et al. (2018) and Timilsena et al. (2022) with the updated results from the present study.

Supplementary Table 4: FAW CLIMEX parameters from Ramirez-Cabral et al. (2017), du Plessis et al. (2018), Timilsena et al. (2022) and the present study.

Supplementary Figure 2: Global maps of climate suitability predictions from Ramirez-Cabral et al. (2017), du Plessis et al. (2018), Timilsena et al. (2022) and the present study.

Supplementary Figure 3: Mapped climate suitability predictions from Ramirez-Cabral et al. (2017), du Plessis et al. (2018), Timilsena et al. (2022) and the present study for sub-tropical climatic areas where fall armyworm is expanding its distribution.

Supplementary Figure 4a: Continental comparison of the percentage of land mass pixels with predicted EI > 0 values.

Supplementary Figure 4b: Regional comparison of the percentage of land mass pixels with predicted EI > 0 values, highlighting areas of expansion of FAW into sub-tropical climates.

Supplementary Figure 4c: Global and selected country level percentages of maize-producing pixels with predicted EI > 0 values.

Supplementary Figure 1. The two variables that contributed most to the first and second principal components used to map S. frugiperda environmental niches. (A) Minimum temperature of coldest week (°C), and (B) Lowest weekly radiation (Wm^{-2})

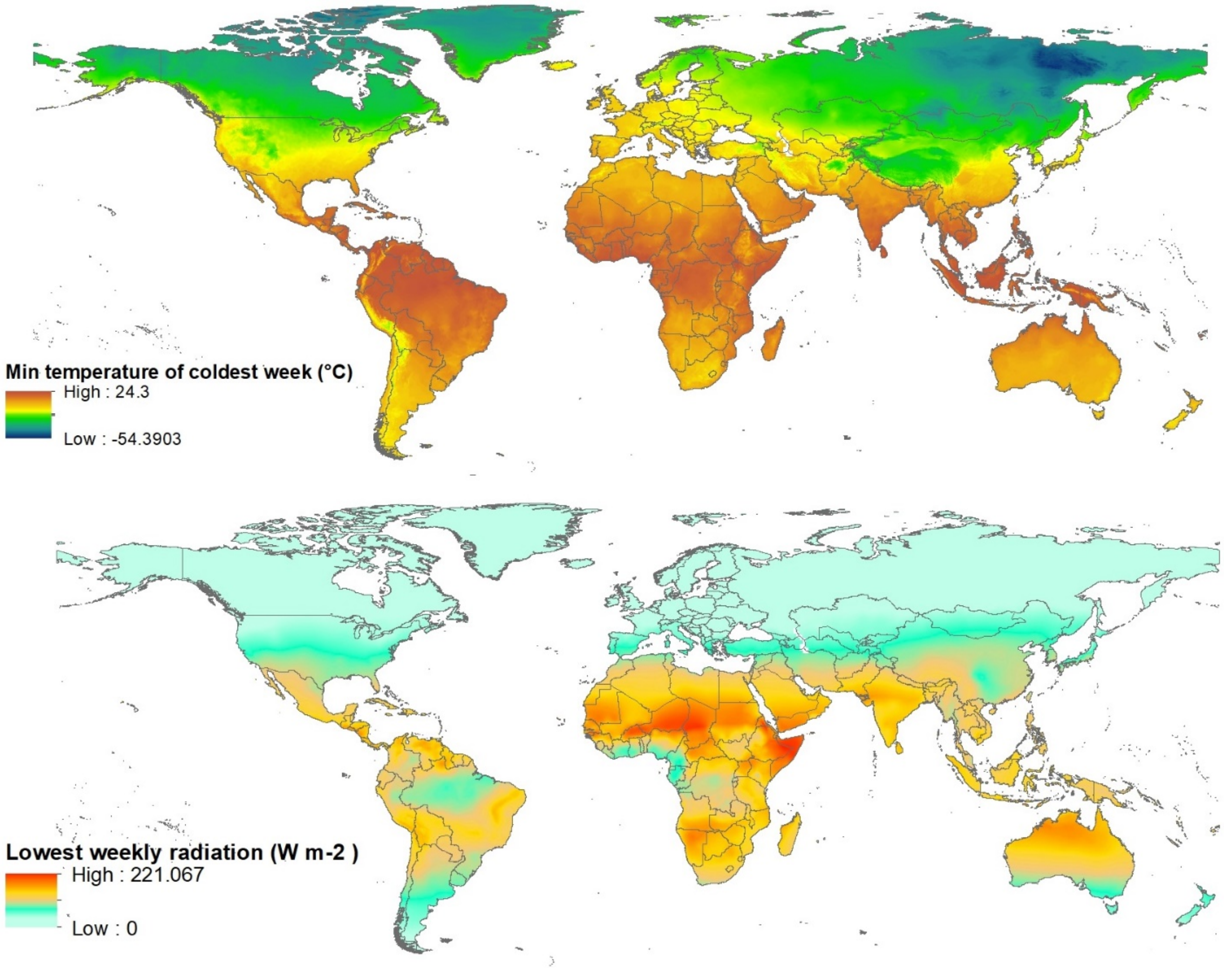

Supplementary Table 1. Pool of bioclimatic variables available for fall armyworm environmental niche modeling prior to variable selection.

No.	Variable Variable Name Dataset	Indicator
01	Annual mean temperature (°C)	Temperature based
02	Mean diurnal temperature range (mean(period max-min)) (°C)	
03	Isothermality (Bio02 ÷ Bio07) *100	
04	Temperature seasonality (C of V)	
05	Max temperature of warmest week (°C)	
06	Min temperature of coldest week (°C)	
07	Temperature annual range (Bio05-Bio06) (°C)	
08	Mean temperature of wettest quarter (°C)	
09	Mean temperature of driest quarter (°C)	
10	Mean temperature of warmest quarter (°C)	
11	Mean temperature of coldest quarter (°C)	
12	Annual precipitation (mm)	Precipitation based
13	Precipitation of wettest week (mm)	
14	Precipitation of driest week (mm)	
15	Precipitation seasonality (C of V)	
16	Precipitation of wettest quarter (mm)	
17	Precipitation of driest quarter (mm)	
18	Precipitation of warmest quarter (mm)	
19	Precipitation of coldest quarter (mm)	
20	Annual mean radiation (W m_{-2})	Solar radiation based
21	Highest weekly radiation (W m_{-2})	
22	Lowest weekly radiation (W m_{-2})	
23	Radiation seasonality (C of V)	
24	Radiation of wettest quarter (W m_{-2})	
25	Radiation of driest quarter (W m_{-2})	
26	Radiation of warmest quarter (W m_{-2})	
27	Radiation of coldest quarter (W m_{-2})	
28	Annual mean moisture index	Moisture based
29	Highest weekly moisture index	
30	Lowest weekly moisture index	
31	Moisture index seasonality (C of V)	
32	Mean moisture index of wettest quarter	
33	Mean moisture index of driest quarter	
34	Mean moisture index of warmest quarter	
35	Mean moisture index of coldest quarter	
36	Elevation (m)	Topography based
37	Slope (deg)	
38	Aspect (deg)	
39	Hillshade	

* Variables without units are dimensionless indices (Kriticos et al. 2012)

Supplementary Table 2. Geographical and other spatial attributes of observed occurrence data.

Region	Observations			Geography	
	Number	Spatially unique with respect to the climate data	Percentage	Native	Invaded
	(count)	*(count)*	*(percent)*		
North America	2,529*	595*	79.6	x	x
Latin America	508	152	16.0	x	
sub-Saharan Africa	110	44	3.5		x
Asia & Pacific	28	18	0.9		x
**Total**	**3,175**	**809**		**933**	**2,242**

Notes: sub-Saharan Africa includes nearby islands. Among the 2,529 total occurrences in North America, 425 of them are from southern Texas and Florida, which falls within the native ranges of the pest. Similarly, 53 of the 595 spatially thinned occurrences in North America also lie within this native range. Thus 2,104 of the total number of occurrences, and 543 of the spatially thinned occurrences in North America fall within the historically invaded, seasonal range.

Supplementary Table 3: FAW CLIMEX parameters developed for the present study.

Parameter	Description	Values	Units
**Moisture**			
SM0	SM0 Lower soil moisture threshold	0.15	
SM1	SM1 Lower optimal soil moisture	0.8	
SM2	SM2 Upper optimal soil moisture	1.5	
SM3	SM3 Upper soil moisture threshold	2.5	
**Temperature**			
DV0	DV0 Lower temperature threshold	8.7	°C
DV1	DV1 Lower optimal temperature	24.6	°C
DV2	DV2 Upper optimal temperature	32.0	°C
DV3	DV3 Upper temperature threshold	39.5	°C
**Cold stress**			
TTCS	TTCS Cold stress temperature threshold	8.70	°C
THCS	THCS Cold stress accumulation rate	-0.001	°C $week^{-1}$
**Heat stress**			
TTHS	TTHS Heat stress temperature threshold	39.5	°C
THHS	THHS Heat stress accumulation rate	0.005	°C $week^{-1}$
**Dry Stress**			
SMDS	SMDS Soil moisture dry stress threshold	0.1	
HDS	HDS Dry stress accumulation rate	-0.005	$week^{-1}$
**Wet Stress**			
SMWS	SMWS Soil moisture wet stress threshold	2.5	
HWS	HWS Wet stress accumulation rate	0.002	$week^{-1}$
**Threshold Annual**			
PDD	PDD Minimum degree day sum needed to	559.00	degree days
**Irrigation Scenario**			
Rainfed	Rainfed (no irrigation)	0.0	mm day^{-1}
Irrigated	Irrigation applied as top-up throughout the	2.5	mm day^{-1}

Notes: Our model parameters drew from the calibrations reported by du Plesis et al. (2018) but were updated to reflect lab evidence on the minimum thermal threshold for FAW coupled with the expanded set of reported occurrence observations used to calibrate our model. The SM (Soil Moisture) index is one of the parameters used to assess pest population growth in CLIMEX. It ranges between 0 and 1. SM=0 indicates no growth, and at SM1 population growth is maximized. SM is a dimensionless index provided to indicate soil moisture content.

Supplementary Section 1: Comparison of Ramirez-Cabral et al. (2017), du Plessis et al. (2018) and Timilsena et al. (2022) with the updated results from the present study.

Here we empirically assess differences between the climatic suitability predictions reported by Ramirez-Cabral et al. (2017), du Plessis et al. (2018), Timilsena et al. (2022), and the present study. To facilitate that, we took the CLIMEX parameters found in Ramirez-Cabral et al. (2017, Table 1), du Plessis et al. (2018, Table1), Timilsena et al. (2022, Table 1), and Supplementary Table 3 (in this document) and re-ran each model using the spatialized climate and crop geography data used for the present study. The resulting global geographic climate suitability maps from these four studies are given in Supplementary Figure 2.

From an initial visual inspection, the overall geographic suitability areas for du Plessis et al. (2018) and Timilsena et al. (2022) are in line with those that we predicted here. However, on closer inspection there are important differences among each of the predictions. Although, Ramirez-Cabral et al. (2017), like du Plessis et al. (2018) and the present study, identify high suitability climes for FAW in the tropical regions of the world, their model overpredicts suitability in temperate areas relative to du Plessis et al. (2018) and the present study. Notably, this overprediction is most evident in areas within European countries, New Zealand, and Southern Australia where Ramirez-Cabral et al. (2017) predicted year-round persistence of FAW in areas that lie outside the reported physiological requirements of fall armyworm. As can be seen in Supplementary Table 4, while Ramirez-Cabral et al. (2017) settled on a lower TTCS (cold stress temperature threshold) relative to du Plessis et al. (2018) and this current study, that enabled them to capture sub-tropical areas into which FAW is expanding, the expanded prediction well into temperate areas could possibly be a reflection of their use of seasonal (transient) populations of fall armyworm in North America to calibrate their model especially in relation with parameters that define optimal FAW thermal requirement. As a result, their predicted EI > 0 areas—representing locations with year-round climate suitability—extended into areas with climates that a) lay well outside the known physiological ranges of FAW, and b) beyond areas with reported sustained presence of the pest. For this reason, we opted to focus the remainder of our comparative assessment on the more comparable EI > 0 results reported by du Plessis et al. (2018) and Timilsena et al. (2022).

du Plessis et al. (2018)'s study (and its CLIMEX parameterization) succeeded in capturing the core tropical distribution of fall armyworm along with some expanded fringes of sub-tropical areas within the reported sustained presence of FAW (like southern border of Florida & Texas in the USA). Timilsena et al. (2022) sought to extend the work of du Plessis et al. (2018) and account for the prospective expansion of the persistent range of FAW associated with climate change. Digging deeper into the differences between the EI > 0 predictions of du Plessis et al. (2018), Timilsena et al. (2022) and the present study, Supplementary Figure 3 provides a closer look at the spatial differences among these three models for selected states (Texas and Florida) and countries (China, Zimbabwe, South Africa and Australia) where FAW is rapidly expanding (Early et al. 2018, Yang et al. 2021, Osabutey et al. 2022). Most of these locations are also areas of potential expansion in Ramasamy et al. (2022)'s FAW climate suitability projection under multiple future climate scenarios.

Supplementary Figure 4, Panel a provides a comparison among Ramirez-Cabral et al. (2017), (du Plessis et al. 2018), (Timilsena et al. 2022) and our model in terms of the percentage of the

worldwide and continent level land masses with predicted values of EI > 0. The data in this figure confirms that Timilsena et al. (2022)'s results are generally in line with those of du Plessis et al. (2018), although they predict a generally expanded footprint for FAW across all the spatial extents tabulated in that figure. Our own estimates expand further on those areas with the climate potential to persistently support FAW populations for reasons we describe in the main body of the paper. The evidence reported in Supplementary Figure 4, Panel b, reinforces the FAW suitability findings across each of the studies, this time for selected countries or U.S. states where FAW is reported to be expanding its footprint.

More specifically, across all the reported regions and countries there is an increase in the predicted climate-suitable areas for FAW persistence when moving from the earlier 2018 (du Plessis et al.) study, to the 2022 (Timilsena et al.) work, and then to the current study. In the case of our study, this reflects the reported (Wu et al. 2022) rapid elastic adaptation of FAW in the cooler end of its habitat. In modeling terms, this is reflected, in part, by the DV0 (lower temperature threshold) value of 8.7°C that we chose, relative to the 12.0 °C value used by du Plessis et al. (2018) and Timilsena et al. (2022). This lowered DV0 value, along with other differences in the parametrization of our model (see Supplementary Table 4), meant our model aligns with both the lab findings of Valdez-Torres et al. (2012) regarding the minimum thermal threshold for FAW on maize and the expanded set of reported occurrence data we used to calibrate our model.

Finally, Supplementary Figure 4c shows the differences among each of the studies in terms of their FAW persistence predictions (i.e., EI > 0) within the geographical extent of maize production reported by You et al. (2014). Ramirez-Cabral et al. (2017) predicts that 70% of the global maize extent is likely to support year-round FAW presence. These global shares are substantially higher than those of du Plessis et al. (2018) who predicted a 51% share, Timilsena et al. (2022) a 54% share and this study 59%. Looking closer at the predicted persistence of FAW in Australia and Zimbabwe we see that Ramirez-Cabral et al. (2017) also predicts much larger FAW persistence shares than the other three studies. Notably, Zimbabwe's predicted persistence share within the area planted to maize is substantial for all four studies. For Australia, du Plessis et al. (2018) and Timilsena et al. (2022) predict persistence shares for maize of 24.1% and 32.4% respectively. Consistent with recent reports,[1,2] and as indicated by Maino et al. (2021) in their Australia specific model, our climatic suitability prediction for FAW in Australia encompasses the locations where FAW is present. The overall share of maize growing pixels that are climatically suitable for FAW is larger, 79%, but well below the 99% predicted by Ramirez-Cabral et al. (2017). Moreover, when cross referenced with the (total land mass) results presented in Supplementary Figure 4b, a sizable share of the FAW persistence we predict for Australia lies outside the geographic extent of maize production.

---

[1] The Biosecurity and Food Safety page of the New South Wales government Department of Primary Industries states the following regarding the spread of FAW in Australia "Fall armyworm is currently present in Australia. It has been found in Queensland, Northern Territory, Western Australia and New South Wales." https://www.dpi.nsw.gov.au/biosecurity/plant/insect-pests-and-plant-diseases/fall-armyworm.

[2] Also see: "The Fall armyworm continuity plan" for the Australian grains industry, v1, November 2020. https://www.planthealthaustralia.com.au/wp-content/uploads/2020/11/Fall-Armworm-Continuity-Plan-2.pdf.

Supplementary Table 4: FAW CLIMEX parameters from Ramirez-Cabral et al. (2017), du Plessis et al. (2018), Timilsena et al. (2022) and the present study.

Parameter	Description	Ramirez-Cabral et al. (2017)	du Plessis et al. (2018)	Timilsena et al. (2022)	This study
Moisture					
SM0	SM0 Lower soil moisture threshold	0.10	0.15	0.15	0.15
SM1	SM1 Lower optimal soil moisture	0.70	0.80	0.80	0.80
SM2	SM2 Upper optimal soil moisture	0.90	1.50	1.50	1.50
SM3	SM3 Upper soil moisture threshold	1.50	2.50	2.00	2.50
Temperature					
DV0	DV0 Lower temperature threshold	12.00	12.00	12.00	8.70
DV1	DV1 Lower optimal temperature	22.00	25.00	25.00	24.60
DV2	DV2 Upper optimal temperature	27.00	30.00	30.00	32.00
DV3	DV3 Upper temperature threshold	34.00	39.00	36.00	39.50
Cold stress					
TTCS	TTCS Cold stress temperature threshold	8.00	12.00	8.00	8.70
THCS	THCS Cold stress accumulation rate	-0.00001	0.001	-0.005	0.00
Heat stress					
TTHS	TTHS Heat stress temperature threshold	38.00	39.00	39.00	39.50
THHS	THHS Heat stress accumulation rate	0.001	0.005	0.0025	0.005
Dry stress					
SMDS	SMDS Soil moisture dry stress threshold	0.10	0.10	0.10	0.10
HDS	HDS Dry stress accumulation rate	-0.001	-0.005	-0.005	-0.005
Wet Stress					
SMWS	SMWS Soil moisture wet stress threshold	1.50	2.50	2.00	2.50
HWS	HWS Wet stress accumulation rate	0.001	0.002	0.01	0.00
Threshold Annual Heat sum					
PDD	PDD Minimum degree day sum needed to complete a generation	559.00	600.00	400.00	559.00
Irrigation Scenario					
	Rainfed (none)	0.00	0.00	0.00	0.00
	Applied as top-up throughout the year	No	2.50	2.50	2.50

Supplementary Figure 2: Global maps of climate suitability predictions from Ramirez-Cabral et al. (2017), du Plessis et al. (2018), Timilsena et al. (2022) and the present study.

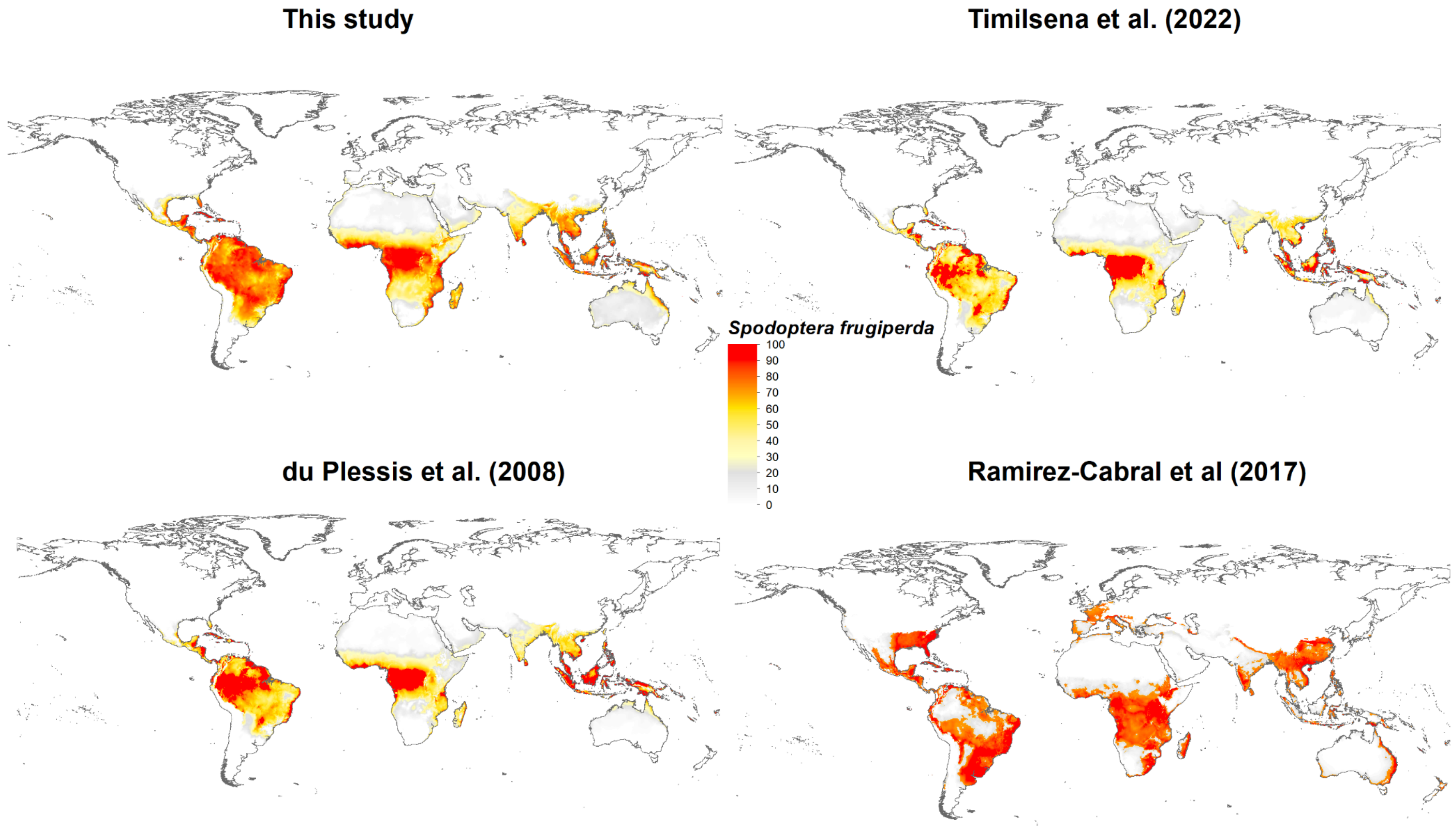

Supplementary Figure 3: Mapped climate suitability predictions from Ramirez-Cabral et al. (2017), du Plessis et al. (2018), Timilsena et al. (2022) and the present study for sub-tropical climatic areas where fall armyworm is expanding its distribution.

(A) Texas and Florida

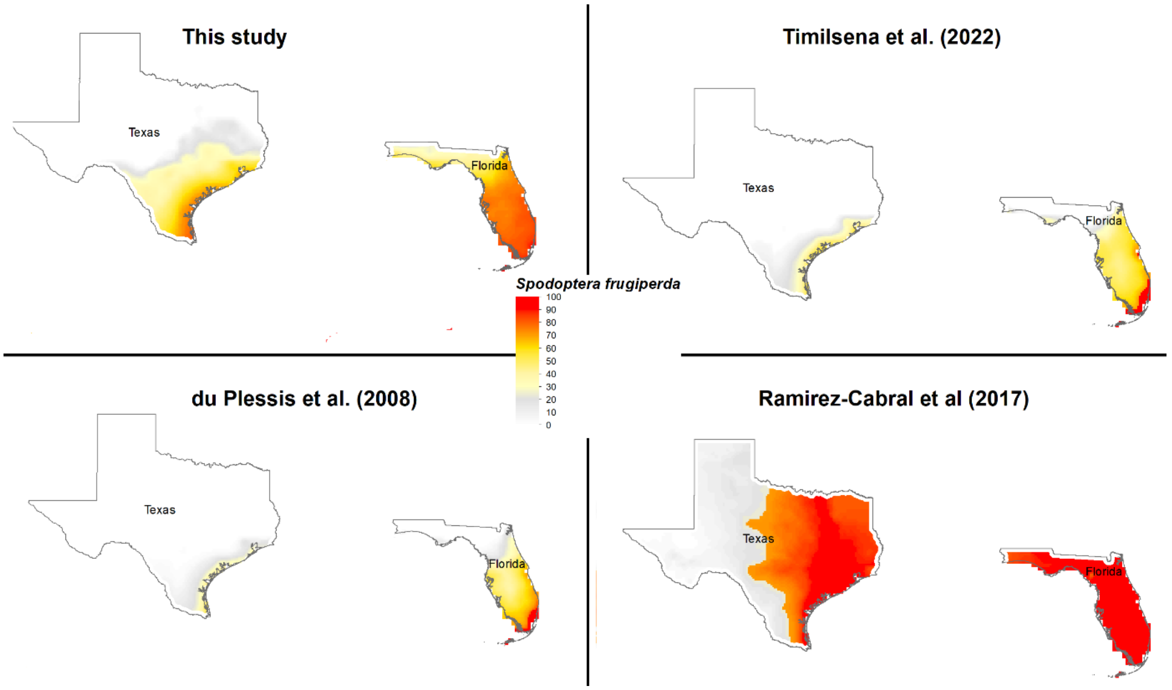

(B) China

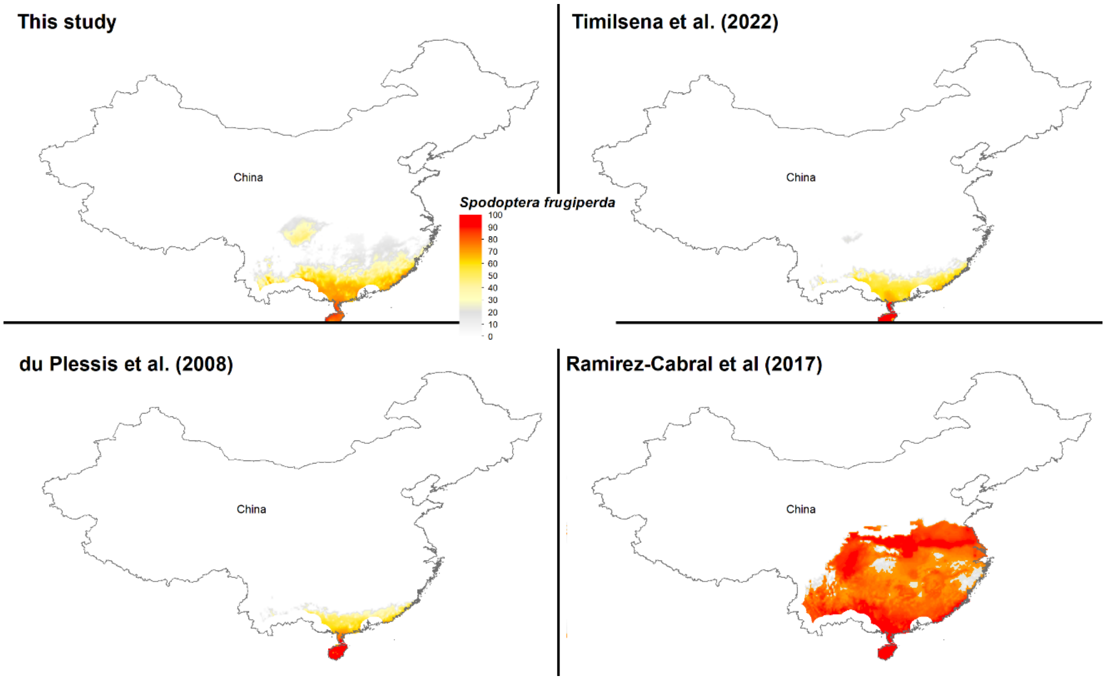

(C) Zimbabwe and South Africa

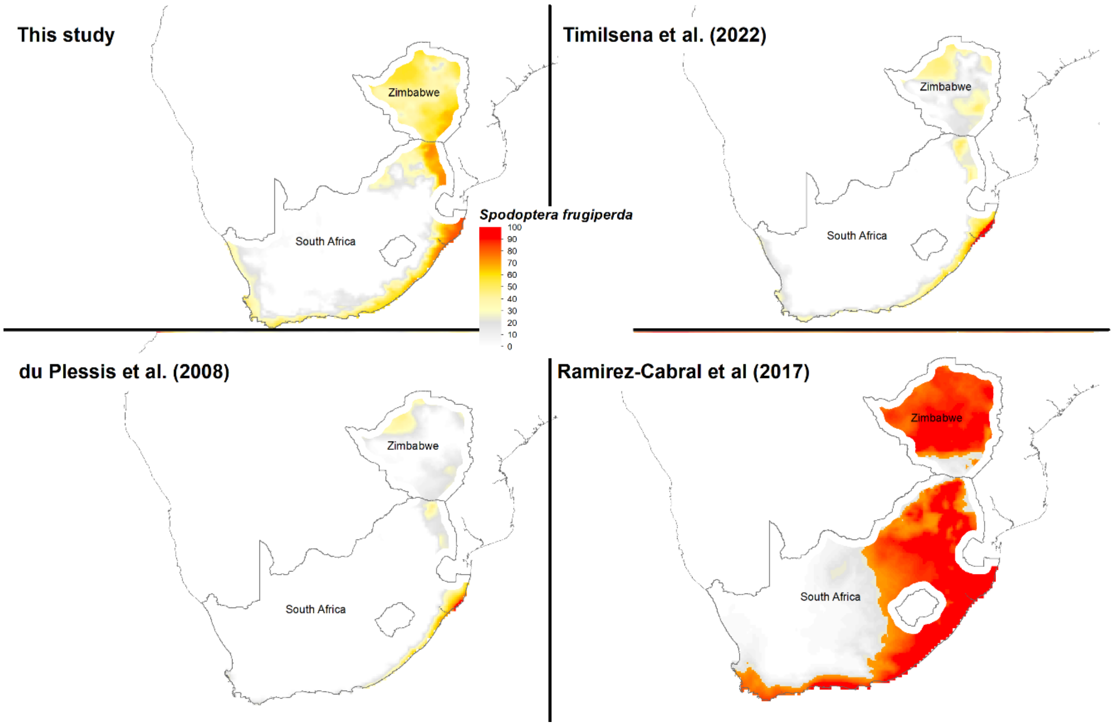

(D) Australia

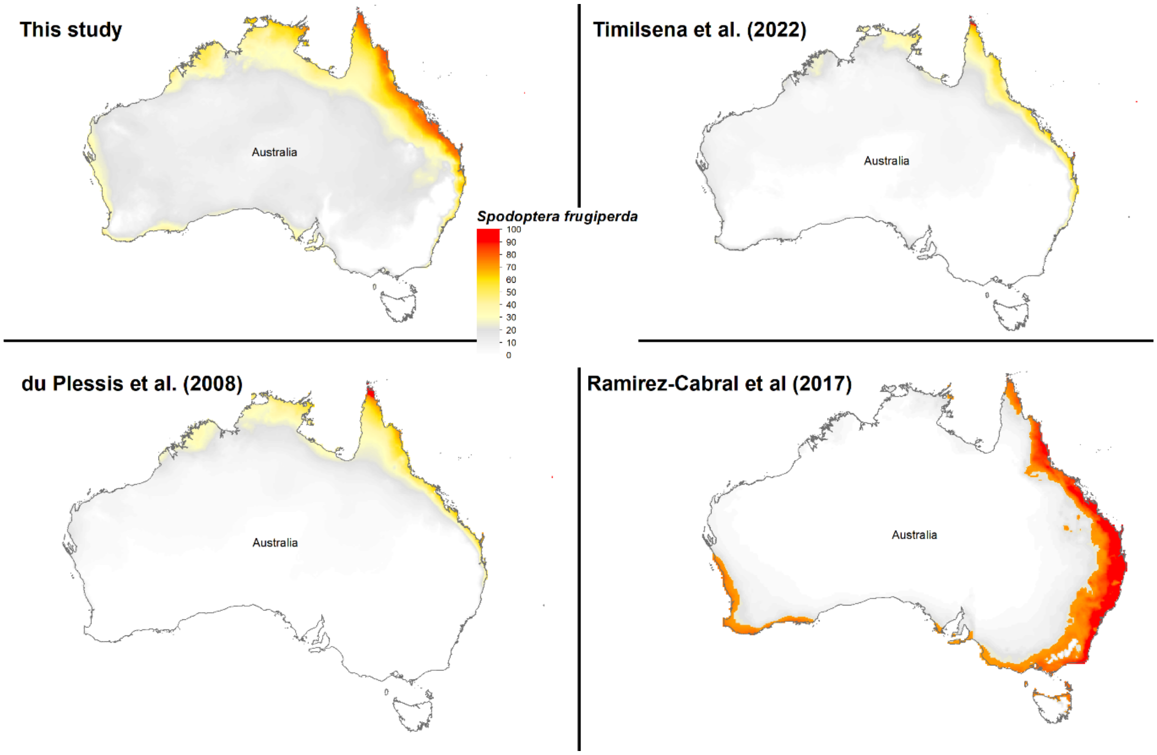

Supplementary Figure 4a: Continental comparison of the percentage of land mass pixels with predicted EI > 0 values.

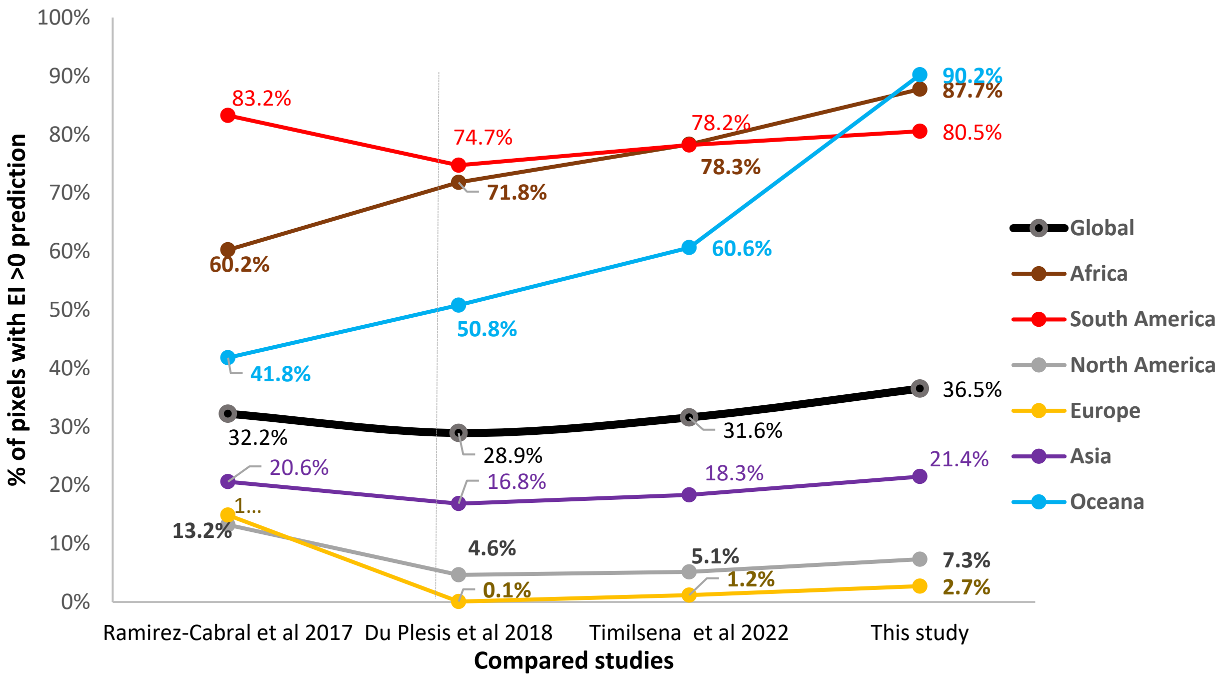

Supplementary Figure 4b: Regional comparison of the percentage of land mass pixels with predicted EI > 0 values, highlighting areas of expansion of FAW into sub-tropical climates.

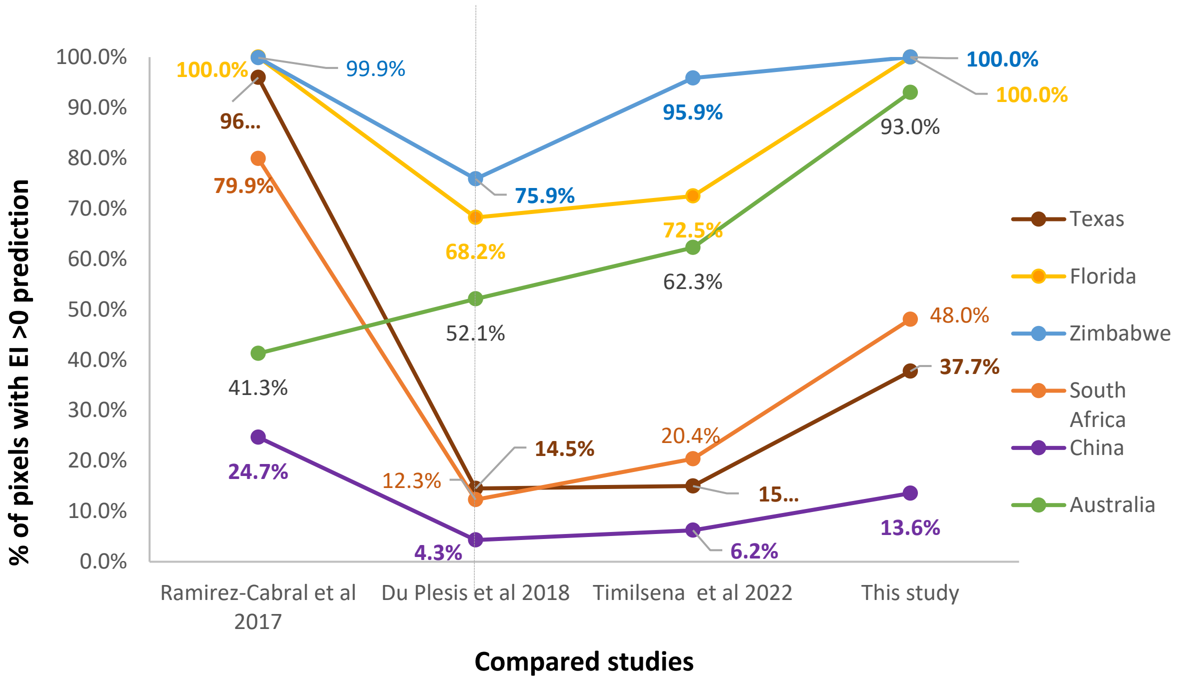

Supplementary Figure 4c: Global and selected country level percentages of maize-producing pixels with predicted EI > 0 values.

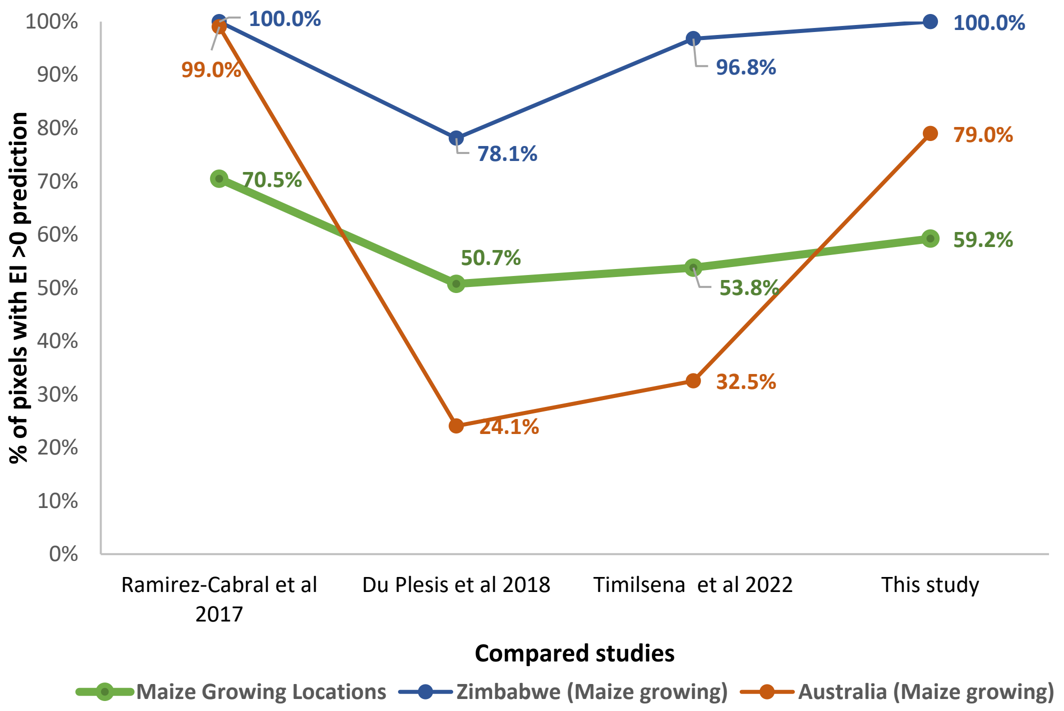

References

du Plessis, H., J. Van den Berg, N. Ota, and D. Kriticos. 2018. Spodoptera frugiperda. Fall Armyworm), CLIMEX modelling. CSIRO-InSTePP Pest Geography.

Early, R., P. González-Moreno, S. T. Murphy, and R. Day. 2018. Forecasting the global extent of invasion of the cereal pest Spodoptera frugiperda, the fall armyworm. NeoBiota **40**:25-50.

Kriticos, D. J., B. L. Webber, A. Leriche, N. Ota, I. Macadam, J. Bathols, and J. K. Scott. 2012. CliMond: global high-resolution historical and future scenario climate surfaces for bioclimatic modelling. Methods in Ecology and Evolution **3**:53-64.

Maino, J. L., R. Schouten, K. Overton, R. Day, S. Ekesi, B. Bett, M. Barton, P. C. Gregg, P. A. Umina, and O. L. Reynolds. 2021. Regional and seasonal activity predictions for fall armyworm in Australia. Current Research in Insect Science **1**:100010.

Osabutey, A. F., B. Y. Seo, A. Kim, T. A. T. Ha, J. Jung, G. Goergen, E. O. Owusu, G.-S. Lee, and Y. H. Koh. 2022. Identification of a fall armyworm (Spodoptera frugiperda)-specific gene and development of a rapid and sensitive loop-mediated isothermal amplification assay. Scientific Reports **12**:1-10.

Ramasamy, M., B. Das, and R. Ramesh. 2022. Predicting climate change impacts on potential worldwide distribution of fall armyworm based on CMIP6 projections. Journal of Pest Science **95**.

Ramirez-Cabral, N. Y. Z., L. Kumar, and F. Shabani. 2017. Future climate scenarios project a decrease in the risk of fall armyworm outbreaks. The Journal of Agricultural Science **155**:1219-1238.

Timilsena, B. P., S. Niassy, E. Kimathi, E. M. Abdel-Rahman, I. Seidl-Adams, M. Wamalwa, H. E. Tonnang, S. Ekesi, D. P. Hughes, and E. G. Rajotte. 2022. Potential Distribution of Fall Armyworm in Africa and Beyond, Considering Climate Change and Irrigation Patterns.

Valdez-Torres, J. B., F. Soto-Landeros, T. Osuna-Enciso, and M. A. Báez-Sañudo. 2012. Phenological prediction models for white corn (Zea mays L.) and fall armyworm (Spodoptera frugiperda JE Smith). Agrociencia **46**:399-410.

Wu, P., M. L. Head, C. Liu, M. Haseeb, and R. Zhang. 2022. The high invasion success of fall armyworm is related to life-history strategies across a range of stressful temperatures. Pest Management Science **https://doi.org/10.1002/ps.6867**.

Yang, X.-m., Y.-f. Song, X.-x. Sun, X.-j. Shen, Q.-l. Wu, H.-w. Zhang, D.-d. Zhang, S.-y. Zhao, G.-m. Liang, and K.-m. Wu. 2021. Population occurrence of the fall armyworm, Spodoptera frugiperda (Lepidoptera: Noctuidae), in the winter season of China. Journal of Integrative Agriculture **20**:772-782.

You, L., S. Wood, U. Wood-Sichra, and W. Wu. 2014. Generating global crop distribution maps: From census to grid. Agricultural Systems **127**:53-60.
